# Computational Identification of Potential RSV L-RdRp Inhibitors with Predicted Superior Activity and Safety Profiles over Remdesivir Using QSAR Modeling, Molecular Docking and Molecular Dynamics Simulations

**DOI:** 10.3390/ph19081142

**Published:** 2026-07-23

**Authors:** Yini Xie, Runqing Jia, Shuo Chen, Fen Li, Guohui Sun

**Affiliations:** 1 Department of Laboratory Medicine, The Fifth People’s Hospital of Huai’an, Huai’an 223300, China; xieyini@emails.bjut.edu.cn; 2 Beijing Key Laboratory of Environmental and Viral Oncology, College of Chemistry and Life Science, Beijing University of Technology, Beijing 100124, China; runqingjia@bjut.edu.cn (R.J.); s202265057cs@emails.bjut.edu.cn (S.C.); 3 Department of Laboratory Medicine, Yangzhou University Affiliated Huai’an Hospital, Huai’an 223300, China; 4 Yangzhou University Huai’an Medical Collaborative Innovation Center, Huai’an 223300, China

**Keywords:** RSV L-RdRp, QSAR, virtual screening, molecular docking, ADMET, molecular dynamics simulations

## Abstract

**Background:** Respiratory syncytial virus (RSV) RNA-dependent RNA polymerase (RdRp) complex is an essential molecular machine for viral genome replication. The L protein, the catalytic subunit of this complex (L-RdRp), is well-characterized structurally and represents a highly promising target for the development of novel small-molecule drugs against RSV. **Methods**: To address the limitations of current QSAR-based virtual screening strategies for RSV L-RdRp inhibitor development, we established a multi-dimensional computer-aided drug screening framework integrating activity, toxicity, drug-likeness, and stability. **Results**: Two OECD-compliant 2D-QSAR models were developed and rigorously validated to predict inhibitory activity and cytotoxicity, respectively. The optimal inhibitory activity model exhibited strong statistical performance, with *R*^2^ = 0.8281, $Q_{LOO}^{2}=$ 0.7653, $R_{test}^{2}=$ 0.8713, $Q_{Fn}^{2}=$ 0.8594 ∼ 0.8837, *CCC*_test_ = 0.9301, *MAE*_test_ = 0.1966. Similarly, the best cytotoxicity model achieved *R*^2^= 0.8263, $Q_{LOO}^{2}$ = 0.7422, $R_{test}^{2}$ = 0.8951, $Q_{Fn}^{2}$ = 0.8108~0.8530, *CCC*_test_ = 0.9081, *MAE*_test_ = 0.1685. Based on these models, a four-step screening workflow—QSAR-based filtering and molecular docking (15,758 → 2446 → 162 → 19 compounds), ADMET evaluation (19 → 5), and molecular dynamics simulations (MDSs)—was implemented to identify promising L-RdRp inhibitors. **Conclusions**: Ultimately, five candidate compounds were selected, all of which demonstrated predicted higher inhibitory activity, lower predicted cytotoxicity, a stable predicted binding mode, and favorable oral bioavailability compared with the reference drug remdesivir. These findings provide valuable in silico-derived lead candidates and a reliable computational workflow for identifying experimental L-RdRp inhibitors targeting RSV.

## 1. Introduction

**Figure 1:**
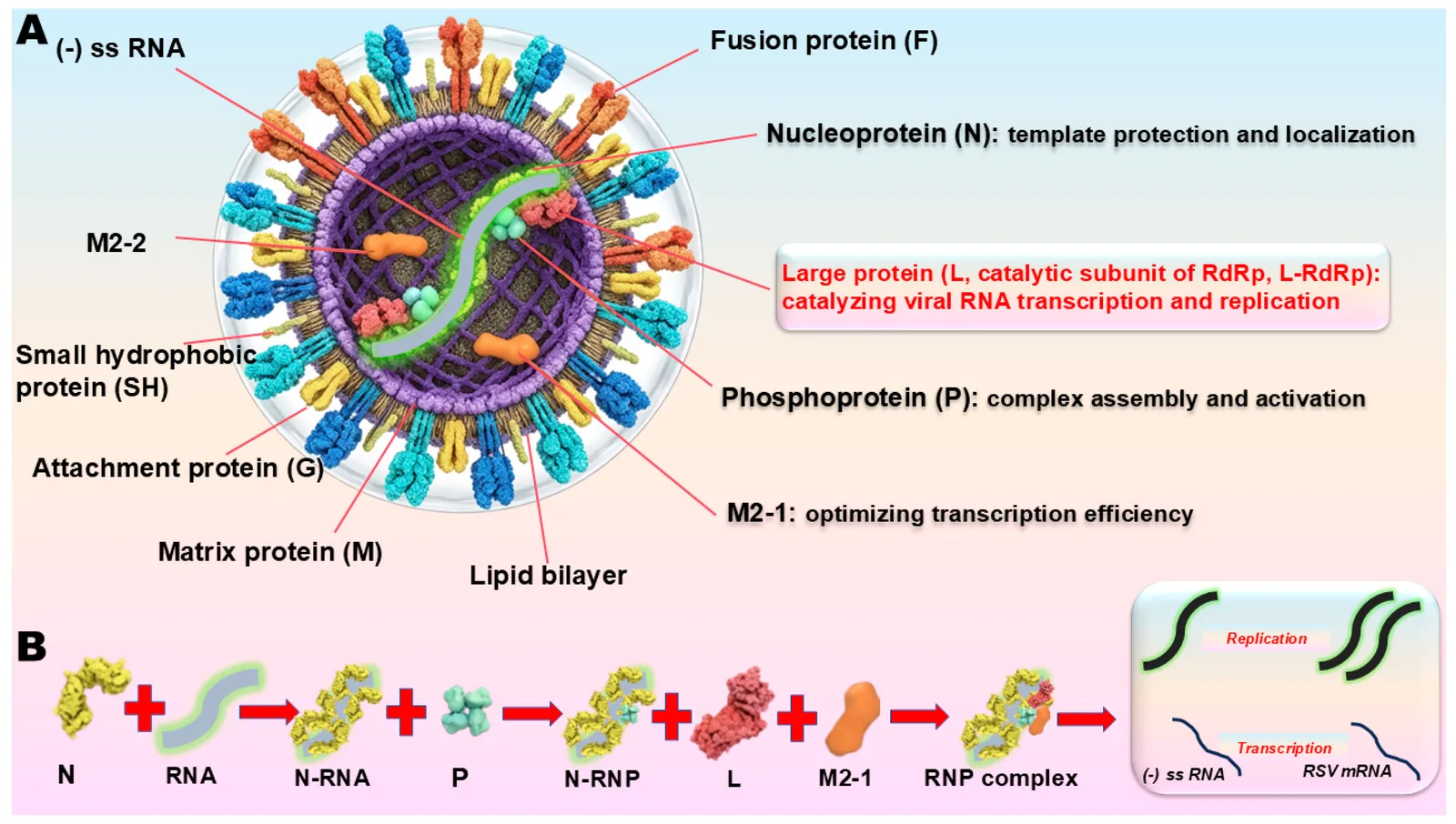
(**A**) The construction of RSV genome; (**B**) assembly process of RSV ribonucleoprotein (RNP) complex and viral RNA synthesis. Nucleoprotein N encapsidates viral genomic RNA to generate N-RNA. N-RNP formed by N-RNA and phosphoprotein P further associates with L protein and recruits transcription anti-terminator M2-1 to assemble functional RNP machinery supporting viral RNA transcription and replication.

Respiratory syncytial virus (RSV) was first isolated in 1955 from chimpanzees with respiratory infections; it has since been identified as one of the primary pathogens responsible for severe lower respiratory tract infections in humans, with a particularly high incidence among infants and young children [1,2]. RSV is an enveloped, single-stranded negative-sense RNA virus. Its genome is approximately 15.2 kb in length and encodes nine structural proteins (Figure 1A), as well as two non-structural proteins [3,4,5,6]. Among them, each structural protein plays an indispensable role in the viral life cycle. The G protein recognizes and binds to host cell receptors, while the F protein mediates membrane fusion to facilitate viral entry [7,8,9,10]. The N protein encapsulates the viral RNA, protecting it from degradation. As a regulator of the L protein, the P protein modulates the enzymatic activity of the L subunit [7,8,9,10]. As the core RNA-dependent RNA polymerase (RdRp), the L protein directly catalyzes the replication and transcription of viral RNA and serves as the primary target for RdRp inhibitors [9,10,11,12]. Furthermore, the M protein is essential for viral particle assembly and release (Figure 1A) [7,8,9,10]. Based on these functional characteristics, current research and development of anti-RSV drugs primarily focuses on three major target categories. The first category targets the F protein, blocking viral entry by inhibiting the conformational changes required for membrane fusion [7]. The second category targets functional domains of viral replication-associated RdRp complex [7,8,9,10,11,12]. The RSV RdRp core complex consists of the multifunctional large protein L and the tetrameric phosphoprotein P and represents the smallest catalytic unit for viral RNA synthesis. For replication and transcription to occur in vivo, the complete RNA synthesis ribonucleoprotein (RNP) complex also requires the participation of nucleoprotein N, which coats the negative-strand genomic RNA, and the transcription anti-termination factor M2-1 (Figure 1B) [9,10]. Among the subunits of the RdRp complex, the L protein serves as the core catalytic component and is the primary target of RdRp inhibitors currently under development [11,12]. The third category targets the N protein; inhibitors of this protein block the progression of the viral life cycle by interfering with key protein-RNA and protein–protein interactions [7,8,9,10].

RSV infection imposes a heavy economic and social burden of disease worldwide. Epidemiological data show that developing countries with limited healthcare resources account for the vast majority of RSV-associated lower respiratory tract infections (LRTIs) cases and deaths [13,14]. In developed countries, infants, the elderly, and immunocompromised individuals remain high-risk groups, being highly susceptible to severe pulmonary infections and even death [15]. Approximately 20–30% of RSV infections in infants and young children progress to severe lower respiratory tract diseases such as bronchitis and pneumonia [16,17]. Compared to influenza patients, elderly individuals with RSV infection have a higher incidence of pneumonia and a significantly increased risk of death within one year of discharge [18].

Although RSV vaccines and prophylactic monoclonal antibodies have been approved for preventive use, no oral specific anti-RSV targeted small-molecule drugs have yet been approved for market release [19]. Clinically, symptomatic and supportive therapy is universally adopted. Although this approach can alleviate symptoms and maintain vital signs in patients with severe LRTI, it cannot block viral replication at its source [11,19]. The use of the broad-spectrum antiviral drug ribavirin may be considered only in immunocompromised individuals with life-threatening severe infections [19,20]. However, due to its teratogenic risk and multiple serious side effects, it is not recommended for routine clinical use [20]. The L protein is the core catalytic subunit of viral replication and represents an ideal target for the development of targeted anti-RSV drugs [21]. Current research on prevention and treatment targeting this protein primarily focuses on the following three major areas: vaccines, monoclonal antibodies, and small-molecule drugs; however, the development of small-molecule inhibitors targeting RdRp has lagged behind, and related studies have not yielded satisfactory results [22]. In recent years, several drug candidates targeting different viral proteins have successively entered the clinical trial phase. Such as RSV RdRp inhibitors, development of PC786 was terminated in Phase II [19,23]; ALS-8176 in infants and young children failed in the Phase II trial [19,24]; and remdesivir continues to undergo Phase II clinical trials for the RSV indication [18,19]. Notably, EDP-323 and S-337395 demonstrated potent antiviral activity and positive efficacy signals in Phase II human viral challenge trials conducted in 2024–2025 [19,25]. However, no selective small-molecule inhibitors targeting viral replication-associated RSV L-RdRp have been officially approved for the clinical treatment of RSV disease. Accordingly, novel potent and low-cytotoxicity L-RdRp inhibitors are urgently required. Traditional drug development model is both time-consuming and resource-intensive, making it difficult to meet the needs of high-throughput screening of large-scale compound libraries [26]. Consequently, the development of efficient and reliable targeted screening strategies has become a critical pathway for identifying novel RSV RdRp inhibitors.

In recent years, computer-aided approaches have assumed an increasingly important role in drug discovery, particularly in the early-stage identification of antiviral lead compounds. Computational modeling and simulation provide detailed information on viral structure, functional dynamics, and molecular mechanisms of virus–host interactions. These approaches support high-throughput virtual screening of compound libraries and facilitate rational target-based drug design. Meanwhile, the 3R principles of animal research ethics, including Reduction, Replacement, and Refinement, have received increasing attention [26,27]. Both the OECD and the U.S. FDA strongly advocate using computer simulations for drug screening and toxicity prediction as a substitute for some animal testing. This approach is particularly suitable during the early stages of testing compounds for toxicity and bioactivity [26,27].

QSAR is among the most commonly applied approaches in virtual screening and lead compound optimization [28]. QSAR models establish mathematical relationships between the physicochemical properties of compounds and their biological activity and can be used to predict the biological activity or potential toxicity of compounds that have not been tested experimentally, thereby facilitating the identification of candidate molecules with enhanced efficacy or reduced toxicity [28]. In practical drug discovery workflows, QSAR is often used in conjunction with other computer-aided drug design methods, such as molecular docking to assess how tightly and how a compound binds to a target; molecular dynamics simulations (MDSs) to further validate the stability of the binding; and pharmacophore analysis and ADMET analysis to determine a compound’s drug-like properties. Combining these methods into a step-by-step screening process can greatly improve the chance of finding safe, effective drug candidates [29]. At present, most QSAR studies on RSV RdRp focus on 3D-QSAR methods. For example, Cox and his colleagues used 3D-QSAR models to improve the structure of GRP-156784 derivatives and enhance their antiviral activity [30]. However, there are still very few 2D-QSAR studies targeting RSV L-RdRp. So far, no research has used 2D-QSAR models to screen large real-world compound libraries. In virtual screening, 2D-QSAR and 3D-QSAR work differently and are suitable for different screening goals. This study selected 2D-QSAR instead of 3D-QSAR for three main reasons. First, the compound library we screened is very large, and 2D-QSAR is faster and uses fewer computing resources. Second, 2D-QSAR descriptors carry definite physicochemical meanings and avoid the uncertainty caused by 3D conformer selection. Third, 2D-QSAR models have better reproducibility and transferability, which supports further application by other researchers [31,32]. Notably, the unified term L-RdRp refers to the catalytic L subunit of the RSV RdRp complex. All tested compounds are defined as L-RdRp inhibitors, which specifically bind the L subunit.

To fill the current lack of 2D-QSAR-based virtual screening methods for RSV L-RdRp inhibitors, this study established a complete in silico screening framework based on interpretable, reproducible and transferable 2D-QSAR. A dataset of small-molecule inhibitors with experimentally verified anti-RSV RdRp activity was collected from public database [30]. Compounds with reliable inhibition and cytotoxicity data were chosen to build two independent 2D-QSAR models that comply with OECD principles. These models can predict antiviral activity and cytotoxicity at the same time, which helps quickly select candidates with balanced efficacy and safety. This study selected remdesivir, a broad-spectrum polymerase inhibitor with well-characterized safety, pharmacokinetics, and polymerase inhibition mechanism, as the control (a non-RSV-specific clinical benchmark) [33].

The framework consists of the following two main parts: first, the construction and evaluation of the model; and second, the use of the model to perform virtual screening of true external compounds. In the first part, we collected molecular structures and bioactivity data for the target of interest to build the model. This included calculating and screening 2D descriptors such as Alva and PaDEL; after splitting the dataset, we performed modeling using a combination of genetic algorithm (GA) and multiple linear regression (MLR). The models were then screened and evaluated using the most stringent validation criteria.

**Figure 2:**
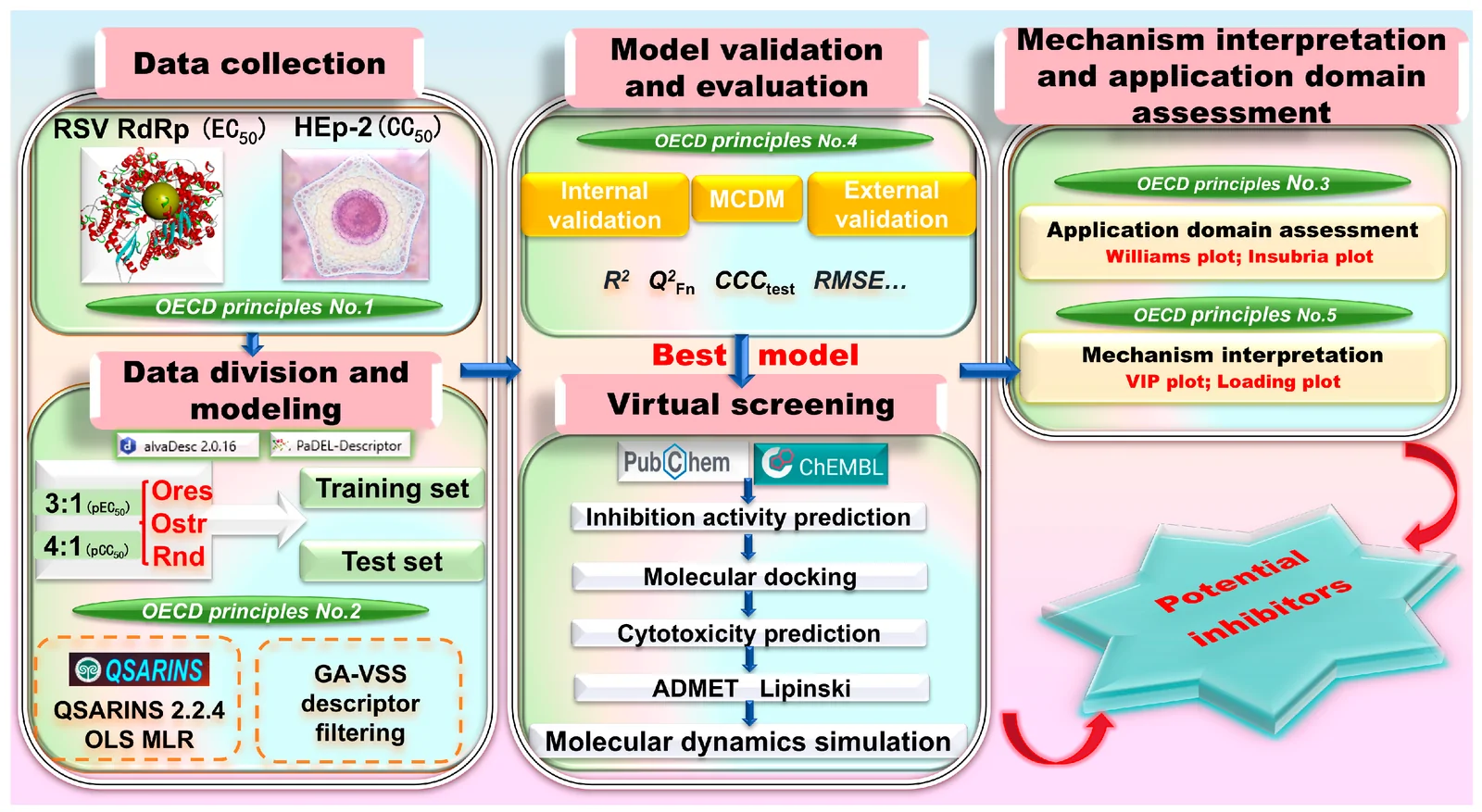
Schematic workflow of the screening of potential RSV L-RdRp inhibitors.

In the second part, the optimized model was used for the virtual screening of potential compounds. By combining molecular docking, drug-likeness rules including Lipinski’s Rule of Five [34,35], and MDS, we identified the most promising lead compounds for experimental validation. Concurrently, we investigated the relationship between the optimized model descriptors and biological activity, mapping molecular descriptors to specific compound structures to provide guidance for further structural optimization of inhibitors. This screening process enables us to comprehensively evaluate potential drug candidates from multiple perspectives. This work provides methodological support and structural references for the discovery and optimization of new RSV L-RdRp inhibitors in antiviral drug development (Figure 2).

## 2. Results

### 2.1. Validation and Selection of the Best QSAR Models

Using strict MCDM validation standards [36], together with internal and external validation indicators [37,38], we identified the most dependable model for predicting inhibitory activity (Rnd, train:test = 3:1) and the optimal cytotoxicity prediction model (Rnd, train:test = 4:1), with detailed model parameters provided in Table 1. For *p*EC_50_-based inhibitory activity prediction model, the main internal validation metrics were *R*^2^ = 0.8281, $Q_{LOO}^{2}=$0.7653, and *R*^2^-$Q_{LOO}^{2}=$ 0.0628; for external validation, the corresponding parameters were $R_{test}^{2}=$ 0.8713, $Q_{Fn}^{2}=$ 0.8594 ∼ 0.8837, ${CCC}_{test}=$ 0.9301, $r_{m}^{2}$ = 0.8144, ${\Delta r}_{m}^{2}=$ 0.0341, ${MAE}_{test}=$ 0.1966, ${RMSE}_{test}$ = 0.2378. For *p*CC_50_-based cytotoxicity prediction model, the main internal validation metrics were *R*^2^ = 0.8263, $Q_{LOO}^{2}=$ 0.7422, and *R*^2^-$Q_{LOO}^{2}=$ 0.0841; for external validation, the corresponding parameters were $R_{test}^{2}=$ 0.8951, $Q_{Fn}^{2}=$ 0.8108 ∼ 0.8530, ${CCC}_{test}=$ 0.9081, $r_{m}^{2}$ = 0.7210, ${\Delta r}_{m}^{2}=$ 0.1292, ${MAE}_{test}=$ 0.1685, ${RMSE}_{test}$ = 0.2120. The internal and external test results of our best prediction models for inhibitory activity and cytotoxicity all satisfy our strictest validation requirements (see detailed modeling information in Supplementary Material File S1, Table S1).

**Figure 3:**
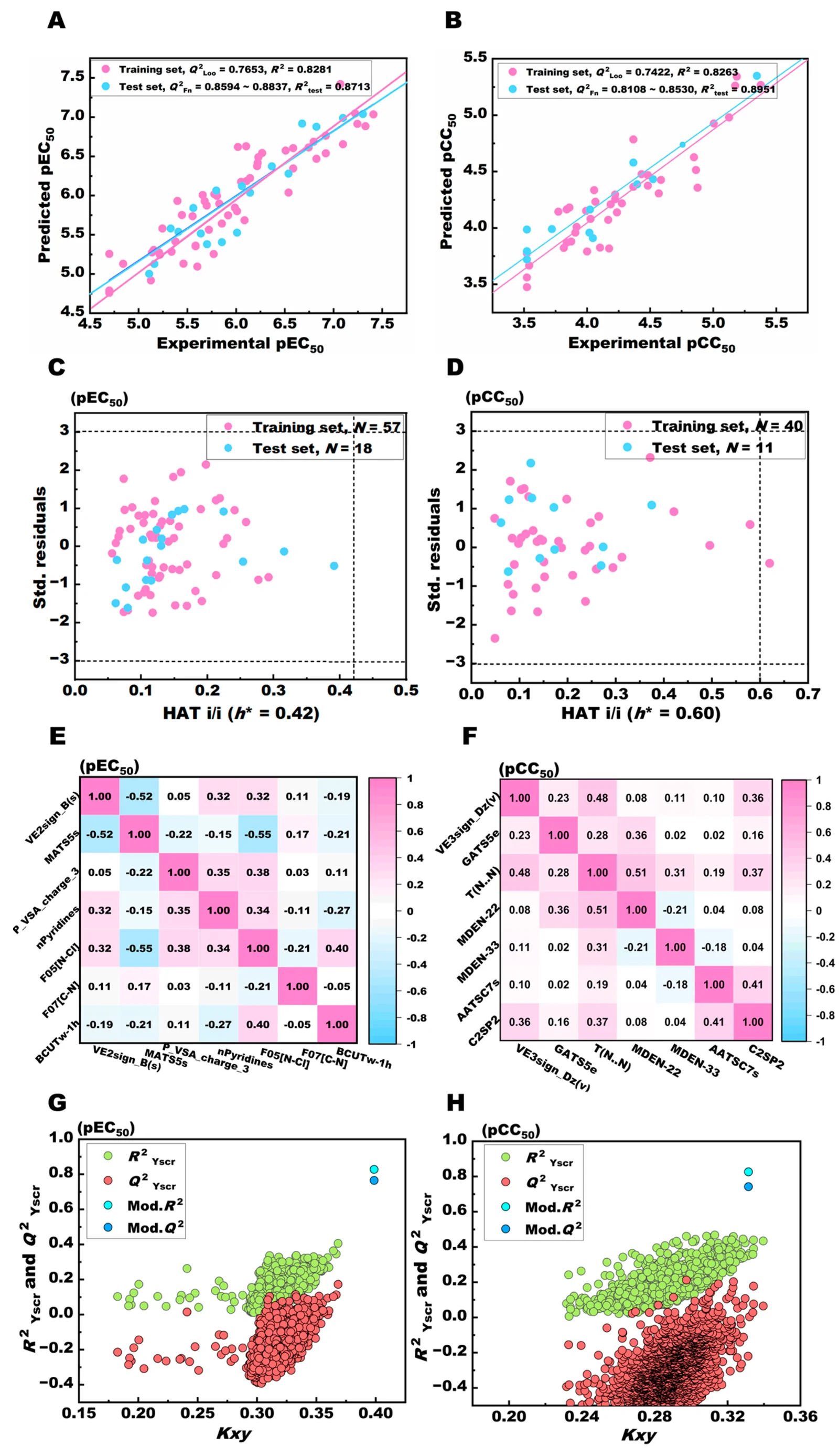
Linear correlation, Williams, intercorrelation and Y-Scrambling plots for the two optimal models. (**A**) Linear correlation plots of predicted vs. experimental values for the optimal inhibitory activity QSAR model; (**B**) those for the optimal cytotoxicity QSAR model. (**C**) Williams plot of the optimal inhibitory activity QSAR model (h* is the leverage threshold); (**D**) that for the optimal cytotoxicity QSAR model (h* is the leverage threshold). (**E**) Intercorrelation plot of seven descriptors in the optimal inhibitory activity QSAR model; (**F**) that for seven descriptors in the optimal cytotoxicity QSAR model. (**G**) Y-Scrambling plot of the optimal inhibitory activity QSAR model; (**H**) that for the optimal cytotoxicity QSAR model.

As shown in the linear regression graphs (Figure 3A,B), the experimental and predicted values for inhibitory activity and cytotoxicity matched very well, with data points tightly clustered around the regression lines and no apparent systematic deviations. The models have high determination coefficients and low RMSE values. These results indicate that the models can clearly show the connection between molecular descriptors and biological activity. The models can thus be used to predict real unknown compounds.

We used Williams plots to check the applicable range of the models. For the *p*EC_50_ model, all training and test compounds had standardized residuals within ±3σ. Their leverage values were also below the threshold. This indicates that the selected samples are highly representative and that the model performs stably and reliably overall. In the *p*CC_50_ model, only one compound in the training set slightly exceeded the h* threshold; upon reviewing its structure, we found that it did not differ significantly from the compounds used for modeling. This observation further confirms the robustness of the cytotoxicity model and excludes overfitting or undue influence from anomalous samples.

The heatmaps for the two models (Figure 3E,F) show low pairwise correlations, ensuring the models’ robustness and interpretability. Furthermore, Figure 3G,H indicate that $Q_{Yscr}^{2}$ and $R_{Yscr}^{2}$ are clearly lower than those of the original model, which proves our results were not obtained by random chance.

### 2.2. Mechanism Explanation of the Optimal Model Descriptors

In accordance with the OECD QSAR guidelines principle No. 5 of “providing mechanistic interpretation whenever possible” [39], this study systematically analyzed the influencing mechanisms of key descriptors in the optimal QSAR models.

**Figure 4:**
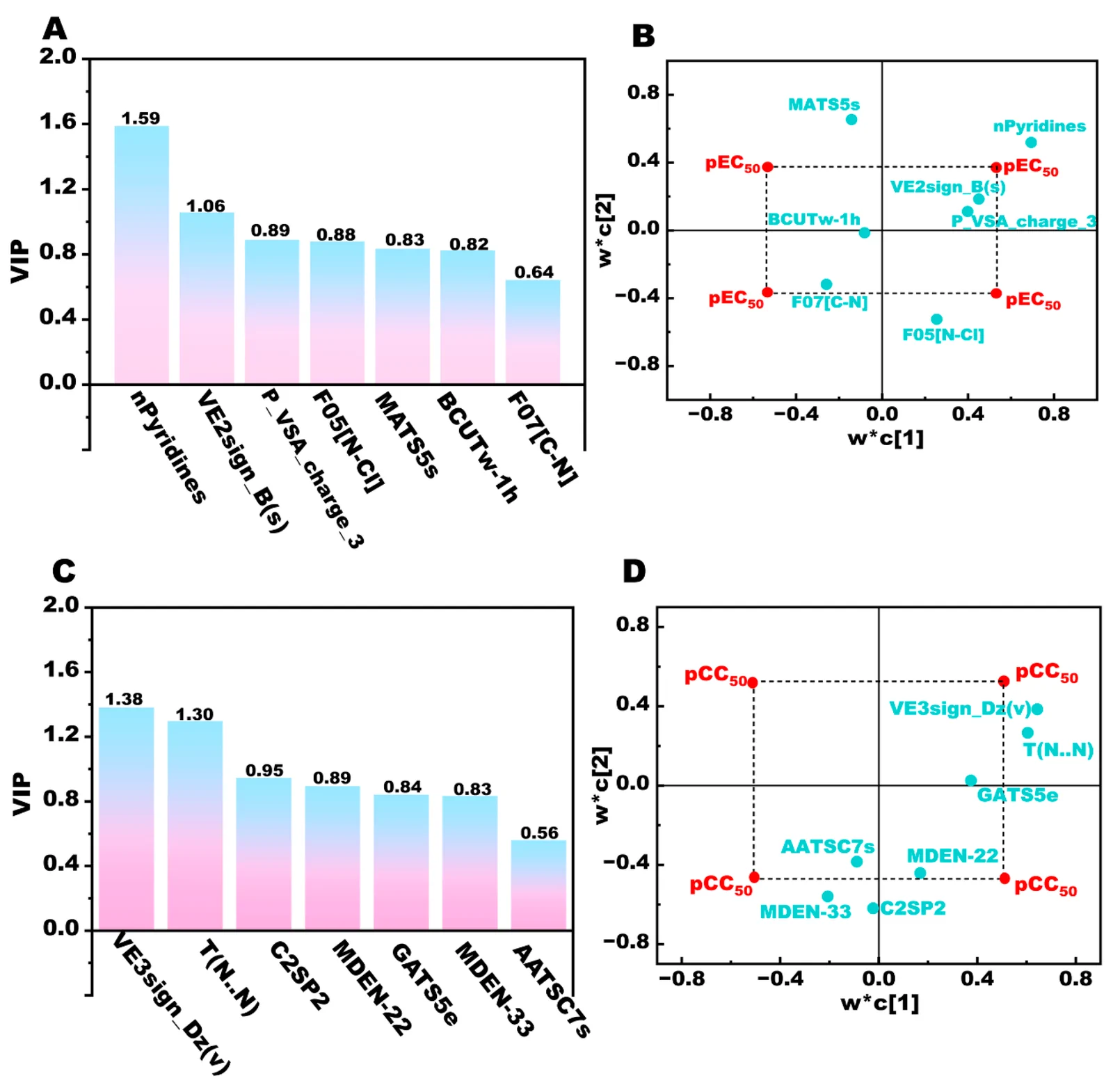
Variable importance and loading plots for the optimal models. (**A**) VIP plot of the inhibitory activity QSAR model; (**B**) loading plot of the seven descriptors in the optimal inhibitory activity QSAR model; (**C**) VIP plot of the cytotoxicity QSAR model; (**D**) loading plot of the seven descriptors in the optimal cytotoxicity QSAR model.

To better understand the role and influence of each descriptor, we carried out Variable Importance in Projection (VIP) analysis and Loading analysis on the two best models for predicting inhibitory activity and cytotoxicity. In the most effective inhibitory activity model (Figure 4A), nPyridines (VIP = 1.59) and VE2sign_B(s) (VIP = 1.06) are significant variables with VIP ≥ 1, indicating their significant contribution to the variation in *p*EC_50_ (inhibitory activity). Descriptors such as P_VSA_charge_3, F05[N-Cl], MATS5s, BCUTw_1h, and F07[C-N] had VIP values < 1, suggesting their role as weaker contributors to the model. Loading plot analysis further confirmed that nPyridines and VE2sign_B(s) are located far from the origin, indicating their stronger explanatory power for inhibitory activity, while the auxiliary descriptors are closer to the origin, reflecting their relatively minor impact on the activity (Figure 4B). In the optimal cytotoxicity prediction model (Figure 4C), VE3sign_D(v) (VIP = 1.38) and T(N..N) (VIP = 1.30) are important descriptors with VIP ≥ 1, showing a dominant role in regulating the *p*CC_50_ (cytotoxicity). C2SP2, MDEN-22, GATS5e, MDEN-33, and AATSC7s had VIP values < 1, functioning as auxiliary descriptors with a lesser impact. In the loading plot, VE3sign_D(v) and T(N..N) were positioned far from the coordinate origin, closely aligned with the *p*CC_50_ response endpoint, indicating their key influence on cytotoxicity (Figure 4D). The remaining auxiliary descriptors were closer to the origin, confirming their secondary regulatory role.

***Mechanistic interpretation of inhibitory activity-related descriptors.*** To further elucidate the mechanism underlying the top-performing inhibitory activity prediction model, we performed a detailed analysis of the seven descriptors included in the model. These descriptors can be categorized into positive correlation and negative correlation groups. The positive correlation descriptors in this model include VE2sign_B(s), MATS5s, P_VSA_charge_3, BCUTw-1h, and nPyridines, while the negative correlation descriptors are F05[N-Cl] and F07[C-N].

**Figure 5:**
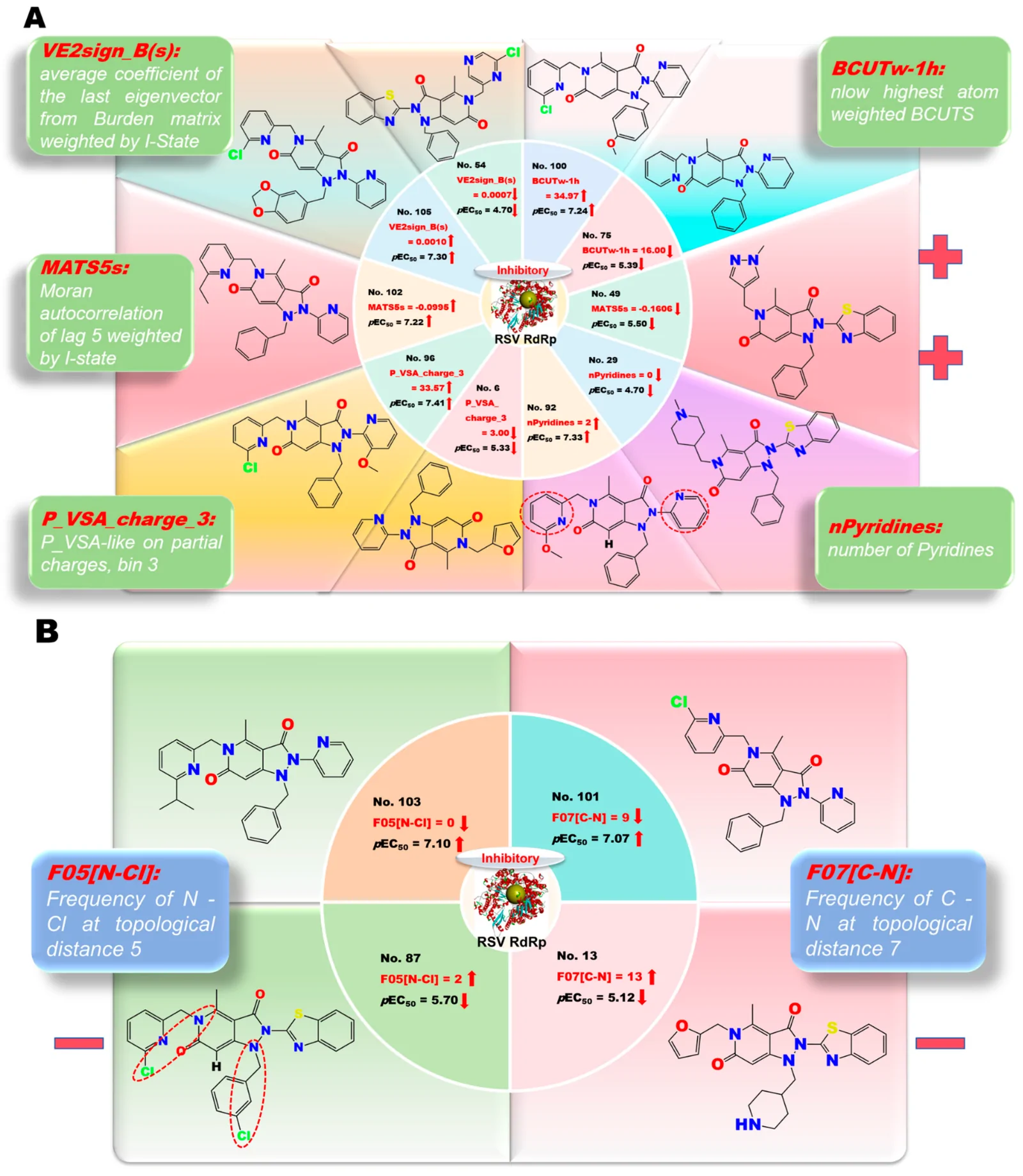
Schematic diagram of the influence of selected descriptors on the response endpoint in the optimal inhibitory activity prediction model. (**A**) Positive descriptors; (**B**) negative descriptors.

Figure 5A illustrates the correlations between positive correlation descriptors and predicted inhibitory activity. VE2sign_B(s) represents the average coefficient of the final eigenvector derived from the Burden matrix, with weighting based on the I-State, and reflects the molecular topological structure and atomic electronic properties [40,41]. For example, compound **105** (VE2sign_B(s) = 0.0010) shows a higher predicted *p*EC_50_ value (7.30) compared to compound **54** (VE2sign_B(s) = 0.0007, *p*EC_50_ = 4.70), which may be associated with compound **105** containing a benzodioxole moiety, which exhibits a higher I-State (electronic activity) in the oxygen atoms compared to the chlorine atoms in compound **54** (Figure 5A). Additionally, complex polycyclic conjugation and diverse bonding types in compound **105** may enhance its inhibitory activity. Within the established QSAR model, higher VE2sign_B(s) values are observed to correlate with elevated predicted inhibitory activity.

MATS5s is a descriptor that describes the Moran autocorrelation of lag 5, with values weighted by I-state, and measures the electronic property correlation of long-range atoms [40,41]. A higher MATS5s value indicates stronger electronic property correlation between atoms separated by five bonds. For instance, compound **102** (MATS5s = −0.0995) shows a higher predicted *p*EC_50_ value (7.22) compared to **49** (MATS5s = −0.1606, *p*EC_50_ = 5.50), which may be correlated with polyazaheterocycles, and conjugated rings in compound **102** strengthen long-range correlations, leading to stronger inhibitory activity (Figure 5A). The results indicate that the higher the MATS5s value, the stronger the inhibitory effect of the compound, which is consistent with the model results.

P_VSA_charge_3 quantifies the atomic volume within a specific charge interval on the molecular surface [40,41]. For example, compound **96** (P_VSA_charge_3 = 33.57) has a higher predicted *p*EC_50_ value (7.41) compared to compound **6** (P_VSA_charge_3 = 3.00, *p*EC_50_ = 5.33). This may be due to compound **96** featuring chlorine- and methoxy-substituted pyridine rings, which significantly enhance the proportion of atomic volume in the third charge interval, thus increasing its inhibitory activity (Figure 5A). In contrast, compound **6** has a more uniform charge distribution, resulting in a lower P_VSA_charge_3 value and is associated with weaker activity.

The descriptor BCUTw-1h is defined as the nlow highest atom weighted BCUTS [40,41] and measures the multi-dimensional electronic-topological characteristics of the molecule [40,41]. For example, compound **100** (BCUTw-1h = 34.97) has a significantly higher predicted *p*EC_50_ value (7.24) compared to compound **75** (BCUTw-1h = 16.00, *p*EC_50_ = 5.39). Compound **100**’s polycyclic conjugation, particularly between chloropyridine and piperidone rings, improves the multi-dimensional structure; thus, it may enhance its inhibitory activity, which is consistent with the model results; a higher BCUTw-1h value commonly signifies stronger molecular inhibitory activity (Figure 5A).

nPyridines is a descriptor used to quantify the number of pyridine rings in a molecule [40,41]. Pyridine rings contribute to inhibitory activity and may be associated with their hydrogen bond-accepting properties and electronic conjugation effects. For instance, compound **92** contains two pyridine rings (*p*EC_50_ = 7.33); these groups can create hydrogen bonds and help keep the molecule firmly bound to the target protein. In contrast, compound **29** lacks pyridine rings, resulting in weaker predicted inhibitory activity (*p*EC_50_ = 4.70). Therefore, the result of the model shows a higher nPyridines value contributes to stronger inhibitory activity (Figure 5A).

Figure 5B shows how descriptors with a negative relationship relate to predicted inhibitory activity. F05[N-Cl] counts the number of N-Cl atom pairs that are five bonds apart in the molecular structure. It reflects specific long-range structures containing nitrogen and chlorine atoms [40,41]. Compound **87** contains two pairs of N-Cl bonds, which may induce steric hindrance or weaken electrostatic interactions, leading to reduced affinity for the L-RdRp binding pocket; consequently, its *p*EC_50_ is only 5.70. Compound **103**, however, does not possess this structural feature, and its *p*EC_50_ value is relatively high, reaching 7.10. This result is consistent with our model analysis and further demonstrates a negative correlation between F05[N-Cl] and inhibitory activity (Figure 5B).

F07[C-N] represents the number of C-N pairs with a topological distance of 7 in the molecule [40,41], reflecting the presence of long-range C-N bonds. Compound **101** contains 9 such structural units; its conformation and electrostatic properties may fit well with the active site of L-RdRp, with a predicted *p*EC_50_ value of 7.07. In contrast, compound **13** contains 13 such structural units; an excessive number of long-range C-N bonds may lead to steric hindrance or poor electrostatic matching, thereby reducing binding affinity to the target, resulting in a predicted *p*EC_50_ of only 5.12. This phenomenon is also consistent with model predictions, indicating that higher F07[C-N] values are associated with lower inhibitory activity, and the two are similarly negatively correlated.

**Figure 6:**
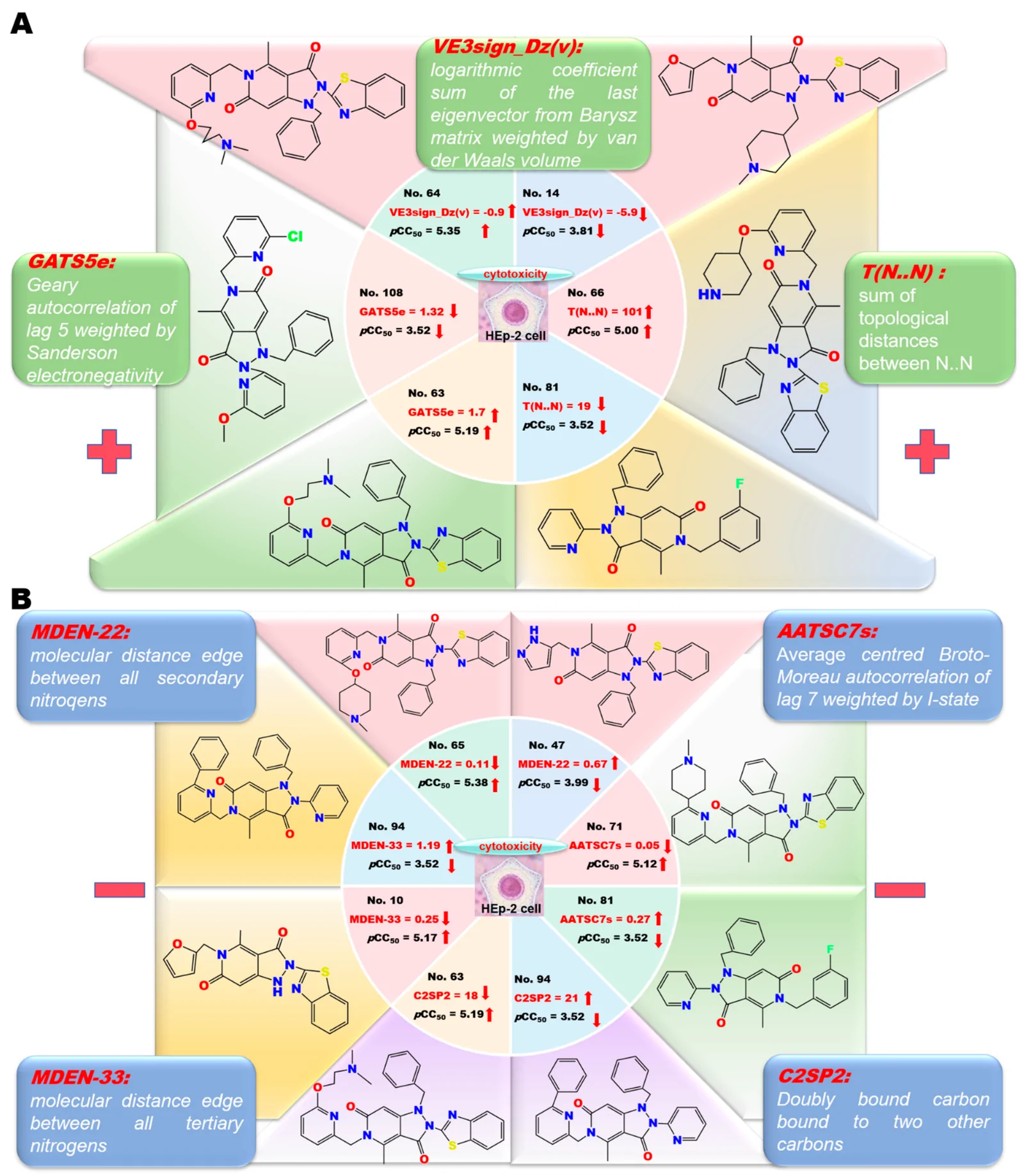
Schematic diagram of the influence of selected descriptors on the response endpoint in the optimal cytotoxicity prediction model. (**A**) Positive descriptors; (**B**) negative descriptors.

***Mechanistic interpretation of cytotoxicity-related descriptors.*** Figure 6A illustrates the molecular descriptors positively correlated with predicted cytotoxicity and their corresponding relationships in the optimal *p*CC_50_ prediction model. Among these, VE3sign_Dz (v) is the sum of the logarithmic coefficients of the final eigenvectors calculated based on the Barysz matrix, weighted by the van der Waals volume; this descriptor integrates the molecule’s structure, electronic distribution, and spatial characteristics [40,41,42]. Compound **64** exhibits a relatively high VE3sign_Dz (v) value (−0.9). Its structure contains a methoxy-substituted pyridine ring and an N-methylmorpholine group. These multidimensional structural features make it more likely to interact strongly with intracellular components, resulting in significant cytotoxicity, with a corresponding predicted *p*CC_50_ value of 5.35. In contrast, compound **14** has a significantly lower VE3sign_Dz (v) value (−5.9). Its complementarity with cells in terms of topological structure, electronic properties, and spatial matching is weaker and may be associated with lower predicted cytotoxicity, with a*p*CC_50_ of only 3.81.

GATS5e represents the lag-5 Geary autocorrelation weighted by Sanderson electronegativity, reflecting the electronegativity similarity and local correlation of atom pairs five bonds apart [40,41,43]. Compound **63** (GATS5e = 1.70), featuring an N,N-dimethylmorpholine ring and a benzothiazole ring, shows enhanced electronic uniformity at this distance and strong predicted cytotoxicity (*p*CC_50_ = 5.19) (Figure 6A). By contrast, compound **108** (GATS5e = 1.32) exhibits weaker electronic correlation and lower predicted cytotoxicity (*p*CC_50_ = 3.52). Thus, increased electronegativity similarity at lag 5 may promote molecular–cell electronic interactions and may be likely to elevate cytotoxicity.

T(N..N) measures the total topological distance between nitrogen atoms and shows how these atoms are distributed in space [40,41]. For example, compound **66** has a high T(N..N) value of 101. Its nitrogen atoms are widely spread and may potentially lead to binding more strongly with cellular targets, resulting in high predicted cytotoxicity with a *p*CC_50_ value of 5.00 (Figure 6A). In comparison, compound **81** has a much lower T(N..N) value of 19. Its nitrogen atoms are concentrated and show lower predicted cytotoxicity with a *p*CC_50_ value of 3.52.

Figure 6B shows the negative correlation between several descriptors and predicted cytotoxicity in the best *p*CC_50_ model. MDEN-22 describes the spatial distribution of secondary nitrogen atoms in the molecule [40,41]. Compound **65** has a low MDEN-22 value of 0.11. Its secondary nitrogen atoms are densely arranged and show strong predicted cytotoxicity with a *p*CC_50_ value of 5.38 (Figure 6B). On the contrary, compound **47** has an MDEN-22 value of 0.67. Its secondary nitrogen atoms are scattered and show lower predicted cytotoxicity with a *p*CC_50_ value of 3.99. It is consistent with model’s result.

MDEN-33, which describes the distribution of tertiary nitrogen atoms, shows a similar pattern [40,41]. Compound **10** has a low MDEN-33 value of 0.25. Its tertiary nitrogen atoms are concentrated and show high predicted cytotoxicity with a *p*CC_50_ value of 5.17. Compound **94** has an MDEN-33 value of 1.19. Its tertiary nitrogen atoms are dispersed and show lower predicted cytotoxicity with a *p*CC_50_ value of 3.52 (Figure 6B). This observed trend is consistent with the outputs of the cytotoxicity QSAR model.

AATSC7s reflects long-range electronic similarity in the molecule [40,41]. Compound **71** has a low AATSC7s value of 0.05. It shows weak electronic correlation and strong predicted cytotoxicity with a *p*CC_50_ value of 5.12. In contrast, compound **81** has a higher AATSC7s value of 0.27. It has more uniform electron distribution and lower predicted cytotoxicity with a *p*CC_50_ value of 3.52. This descriptor is negatively correlated with cytotoxicity (Figure 6B). Within the cytotoxicity QSAR model, this descriptor exhibits a negative statistical correlation with predicted cytotoxicity.

C2SP2 measures the number of double-bonded carbon units and reflects molecular unsaturation, rigidity and hydrophobicity [40,41]. Compound **63** yields a C2SP2 value of 18; it possesses a balanced level of rigidity and flexibility and corresponds to higher predicted cytotoxicity (*p*CC_50_ = 5.19). Compound **94** exhibits a C2SP2 value of 21 and displays greater molecular rigidity, accompanied by lower predicted cytotoxicity (*p*CC_50_ = 3.52) (Figure 6B). Within the applicability domain of the developed model, higher C2SP2 values are observed to be statistically associated with reduced predicted cytotoxicity.

This study further investigated the molecular features corresponding to key descriptors, as well as their intrinsic relationships with structure–activity and structure–cytotoxicity relationships. Finally, we identified several potential compound fragments with strong predicted inhibitory activity and low predicted cytotoxicity, which may offer clear guidance for the structural optimization of RSV L-RdRp inhibitors.

Within the established QSAR models, to make these compounds more effective at inhibiting the target, structural optimization should focus on positive descriptors. More nitrogen-containing heterocycles can be introduced into the molecular framework, such as adding 1–2 pyridine rings, to utilize the nitrogen atoms on the rings as hydrogen-bonding sites, thus possibly helping the compound bind more strongly to the active site of RSV L-RdRp. Furthermore, constructing a rigid polycyclic conjugated framework can optimize parameters such as VE2sign_B(s) and BCUTw-1h, potentially enabling more stable conformational binding of the small molecule to the target. The targeted introduction of highly electronegative groups, such as chlorine atoms, can also modulate the P_VSA_charge_3 and MATS5s parameters might be beneficial to binding affinity. However, it is essential to avoid detrimental structures, particularly the F05[N-Cl] structure and an excessive number of F07[C-N] structures. Such groups can easily cause steric hindrance or disrupt electron pair balance and may reduce the compound’s binding capacity and antiviral inhibitory activity.

To reduce the cytotoxicity of the compound, relevant structural factors must be strictly controlled as follows: avoid overly complex molecular topologies as much as possible and reduce the number of flexible moieties to lower the VE3sign_Dz(v) value; concentrate nitrogen atoms to minimize the T(N..N) value; simultaneously reduce molecular electronegativity similarity to avoid excessively high GATS5e values, thereby minimizing non-specific binding between the compound and cells. Additionally, low cytotoxicity structural features can be enhanced by appropriately separating secondary and tertiary nitrogen atoms and optimizing the MDEN-22 and MDEN-33 parameters; increase the AATSC7s value to ensure a more uniform electronic distribution; control C2SP2 within an appropriate range to balance molecular rigidity with various physicochemical properties.

Overall, the QSAR model developed in this study enables efficient prediction of a compound’s antiviral activity and provides a statistically grounded framework for the rational structural optimization of lead compounds. Based on the screened key descriptors, this study has summarized a structural optimization strategy for RSV L-RdRp inhibitors as follows: an ideal compound should possess a highly rigid polycyclic conjugated core skeleton, contain 1–2 pyridine rings, and incorporate suitable electrophilic substituents, while avoiding all unfavorable topological structures and achieving an optimal balance between nitrogen atom distribution and molecular rigidity. This strategy translates abstract QSAR descriptors into intuitive and actionable molecular modification guidelines based on the statistical trends obtained from the model, providing a reliable theoretical framework for the design and development of future RSV L-RdRp inhibitors with high inhibitory activity and low cytotoxicity.

### 2.3. First Screening with the Optimal Activity QSAR Model

**Figure 7:**
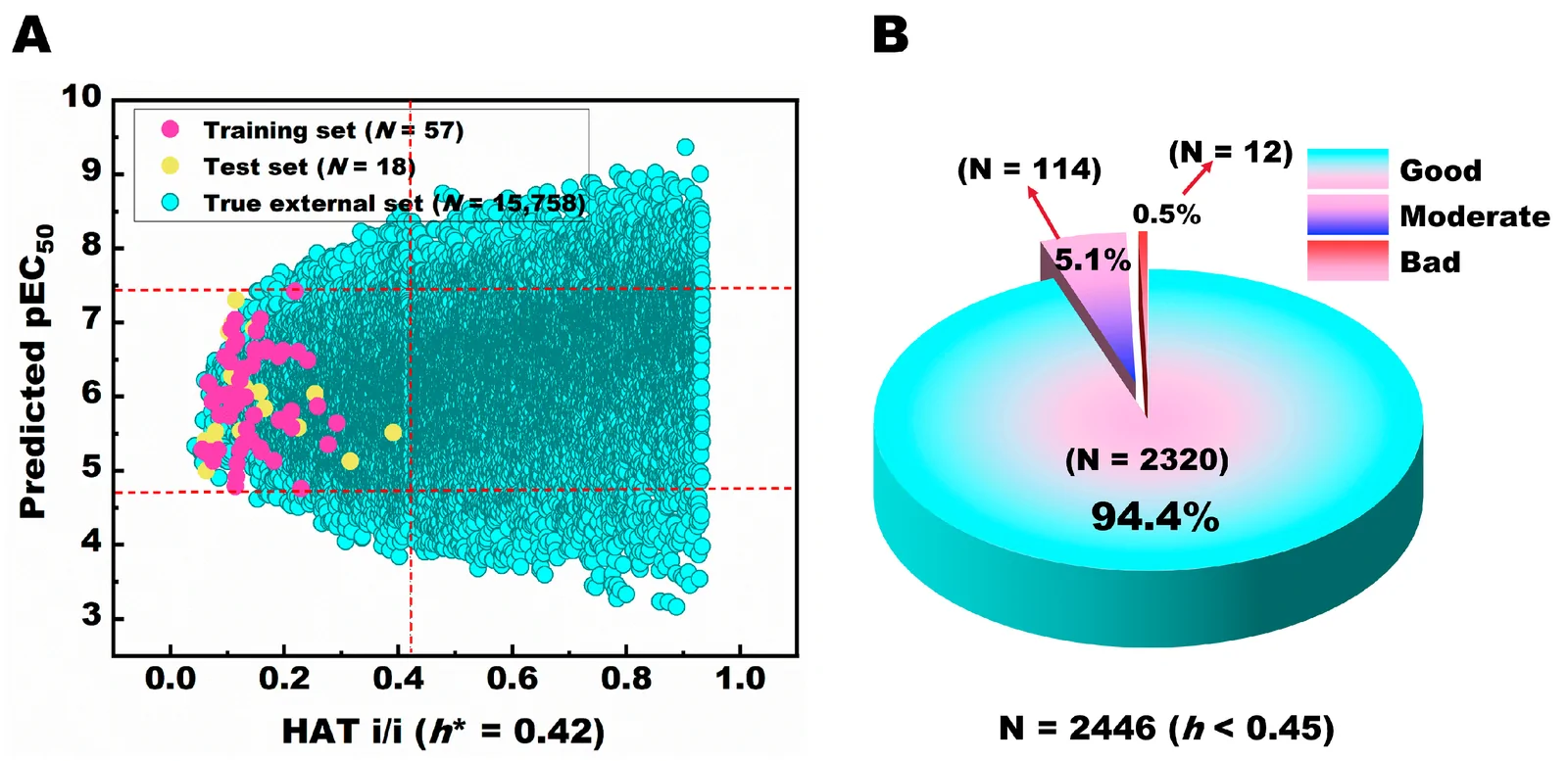
Prediction outcomes for untested external compounds. (**A**) Insubria plot and (**B**) PRI results for 15,758 untested external compounds from the PubChem database (h* is the leverage threshold).

After constructing the optimal QSAR model, it was applied to the virtual screening of real-world external compounds with the aim of identifying RSV L-RdRp inhibitors that exhibit higher inhibitory activity than remdesivir and lower cytotoxicity. First, the *p*EC_50_ prediction model was used to estimate the activity of a total of 15,758 external compounds from the ChEMBL and PubChem databases, and the reliability of the predictions was evaluated using an Insubria plot based on leverage values and predicted *p*EC_50_ (Figure 7A). The results showed that for compounds with leverage values within a reasonable range, the distribution of predicted activity was highly consistent with that of the training and test sets, indicating that the predictions were reliable; compounds exceeding the leverage threshold were considered outliers. The following dual screening strategy was adopted: (i) a leverage threshold of *h* < 0.45 was applied, slightly relaxed relative to the model warning limit (h* = 0.42) to preserve structural diversity and avoid excluding novel chemotypes; and (ii) predicted inhibitory activity exceeding that of Remdesivir (*p*EC_50_ > 7.40). Compounds failing either criterion were excluded, resulting in 2446 candidate molecules retained for further analysis (Supplementary Material File S1, Table S2).

A PRI assessment was performed on these 2446 real-world external compounds. The results showed that 94.4% were classified as having “good” predictive reliability, 5.1% as “moderate,” and only 0.5% as “poor” (Figure 7B), indicating that this inhibitory activity model possesses strong predictive reliability (Supplementary Material File S1, Table S3).

**Figure 8:**
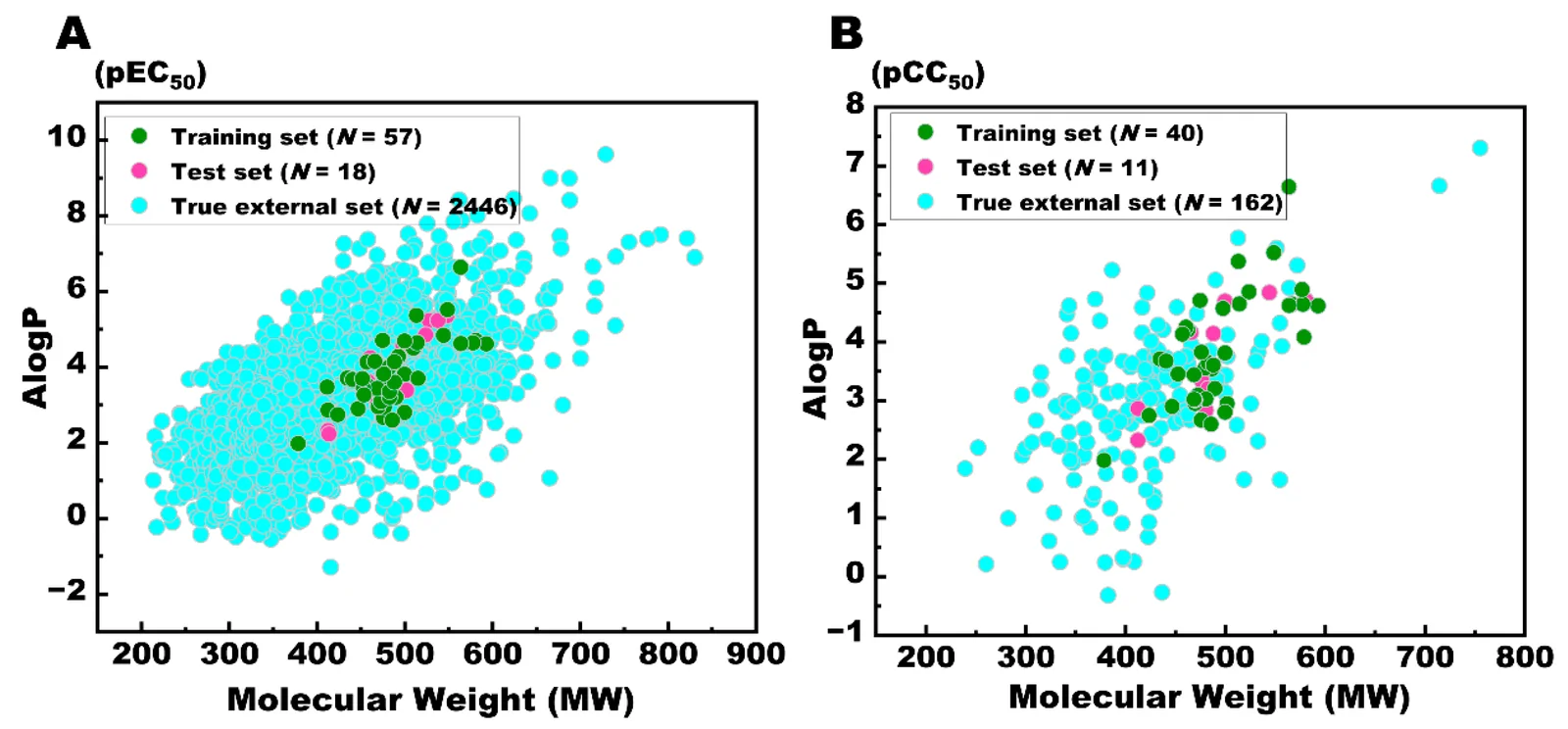
Chemical space distribution. (**A**) Training compounds and actual external compounds for the best activity prediction model; (**B**) training compounds and actual external compounds for the best cytotoxicity model (N = 162: compounds filtered by the optimal activity model and molecular docking, then assessed by the cytotoxicity model).

Finally, chemical space coverage was analyzed for the inhibitory activity (*p*EC_50_) model (Figure 8A). For the *p*EC_50_ model, training compounds were mainly distributed within a drug-like region (MW = 400–600, AlogP = 2–6), with the test set showing substantial overlap, supporting the validity of model generalization. The real external compounds covered a broader chemical space (MW = 200–800, AlogP = −2–10) while still overlapping significantly with the core regions of the training and test sets, indicating a wide and reliable AD. Notably, a subset of compounds exhibited very high lipophilicity (AlogP > 6) or large molecular weight (MW > 600). Although these compounds fell within the applicability domain (AD) of the inhibitory activity prediction model, their inhibitory activity represents merely computational predictions and requires thorough experimental validation in subsequent biological evaluations.

### 2.4. Analysis of Molecular Docking Results

Based on the optimal inhibitory activity QSAR model, 2446 compounds located within the model’s AD and predicted to exhibit higher predicted inhibitory activity than Remdesivir (*p*EC_50_ = 7.40) were selected for structure-based validation. We then performed molecular docking between these compounds and the RSV L-RdRp allosteric pocket (PDB ID: 8FPI), with remdesivir as a control. The results showed that all selected compounds bind well to the L-RdRp allosteric pocket and more favorable predicted intermolecular interactions. Among them, the CDOCKER_INTERACTION_ENERGY of remdesivir was −57.55 kcal/mol, and 162 external candidate compounds had docking energies below −45 kcal/mol, indicating that they can form stable and strong interactions with the L-RdRp allosteric pocket (Supplementary Material File S1, Table S4). Molecular docking serves as a further validation of the QSAR virtual screening. It not only illustrates the plausible binding modes of small molecules against the target RSV L-RdRp protein but also offers qualitative references to screen ligands with more favorable predicted static intermolecular contacts, laying a basis for subsequent druggability evaluation and molecular dynamics trajectory analysis.

### 2.5. Screening with the Optimal Cytotoxicity QSAR Model

We then used an optimal cytotoxicity prediction model to predict the cytotoxicity risk of remdesivir and 162 compounds that showed good intermolecular interactions in the molecular docking results, in order to exclude highly cytotoxic candidate compounds after docking and improve the druggability of the retained molecules. The predicted *p*CC_50_ value for remdesivir was 6.31; the complete statistical distribution of the predicted *p*CC_50_ values for all 162 compounds is summarized in the Supplementary Materials to reflect the overall cytotoxicity profile of this compound library (Supplementary Material File S1, Table S5). To validate the reliability and applicability of this cytotoxicity model, we plotted a chemical space distribution map (Figure 8B). The results showed that the training set was primarily concentrated in the drug-like region with molecular weights of 400–600 and AlogP values of 3–5. The distribution of the test set highly overlapped with that of the training set, indicating strong generalization ability and stable, reliable predictions. Furthermore, most external compounds were distributed in the molecular weight range of 300–600 and AlogP 2–6, which significantly overlaps with the core chemical space of the modeling dataset, further confirming the model’s broad applicability. Ultimately, cytotoxicity prediction screening identified 19 lead compounds with *p*CC_50_ values lower than that of remdesivir (6.31) (Supplementary Material File S1, Table S6). All *p*CC_50_ values in this study are purely computational predictions from the QSAR model and are not experimentally measured CC_50_ data from cell experiments; the actual cytotoxic effects of the screened molecules still require further validation through subsequent in vitro cell assays.

### 2.6. ADMET Analysis of Candidate Compounds

We conducted ADMET drugability assessments on the 19 compounds identified through screening and ultimately identified five candidate compounds with greater overall development potential than remdesivir (See details in Supplementary Material File S2).

**Figure 9:**
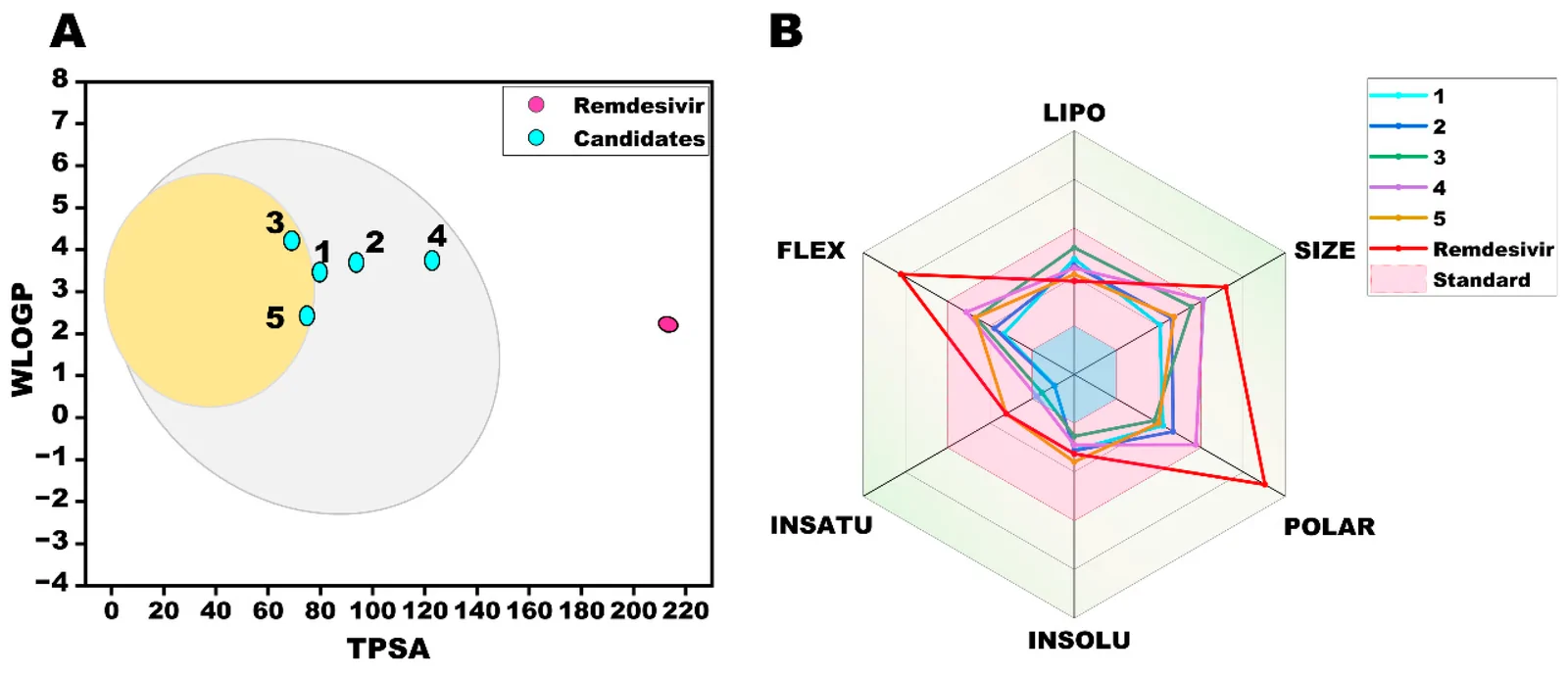
(**A**) Boiled egg plot. (**B**) Radar plot of five candidates: (1) **122478454**, (2) **166175914**, (3) **129032008**, (4) **70798870**, and (5) **134450581**.

The BOILED-Egg plot (Figure 9A) shows that compounds **122478454**, **166175914**, and **70798870** exhibit good gastrointestinal absorption properties, while compounds **129032008** and **134450581** demonstrate both high gastrointestinal absorption rates and excellent blood–brain barrier permeability. In contrast, remdesivir falls within the non-permeable region and exhibits weak passive gastrointestinal absorption, consistent with its clinical intravenous administration route; radar chart analysis (Figure 9B) further confirms that these five candidate compounds fall within the ideal drug-like range for all six key physicochemical properties, whereas remdesivir exhibits significant deviations in molecular size, polarity, and flexibility.

Detailed ADMET prediction results (Table 2) indicate that the human intestinal absorption rates of the five candidate compounds all exceed 90%, far higher than Remdesivir’s 71.3%. Furthermore, they exhibit superior Caco-2 permeability and stronger oral absorption potential, while their skin permeability is comparable to that of Remdesivir. In terms of in vivo distribution, compounds **122478454** and **70798870** have higher free drug fractions, which helps increase their effective concentrations in the body. In terms of metabolism, compounds **134450581** and **122478454** do not inhibit major CYP enzymes in humans; therefore, they are less likely to cause drug–drug interactions compared to remdesivir. All candidate compounds are expected to have relatively rapid clearance rates in the body. Regarding safety, compound **129032008** poses no risk of hepatotoxicity; meanwhile, compound **122478454** does not inhibit the hERG channel, resulting in a lower risk of cardiotoxicity. No mutagenicity was predicted for any candidate. In comprehensive evaluation using Lipinski, Ghose, Oprea, Veber, and Muegge rules, all five compounds meet at least three criteria. This makes them more druglike than remdesivir.

In conclusion, these five candidates overcome remdesivir’s main drawback of poor oral absorption. They also show favorable distribution, metabolism, and safety profiles. With intestinal absorption over 90% and Caco-2 permeability 1.5 to 3 times higher than remdesivir, they have strong potential for oral delivery. This could greatly improve patient compliance and reduce clinical costs. Importantly, these compounds maintain inhibitory activity similar to or better than remdesivir. They therefore represent promising orally available next-generation RSV L-RdRp inhibitors with improved druggability.

### 2.7. Visualization of Docking Results for Remdesivir and the Five Candidate Compounds with L-RdRp (8FPI)

**Figure 10:**
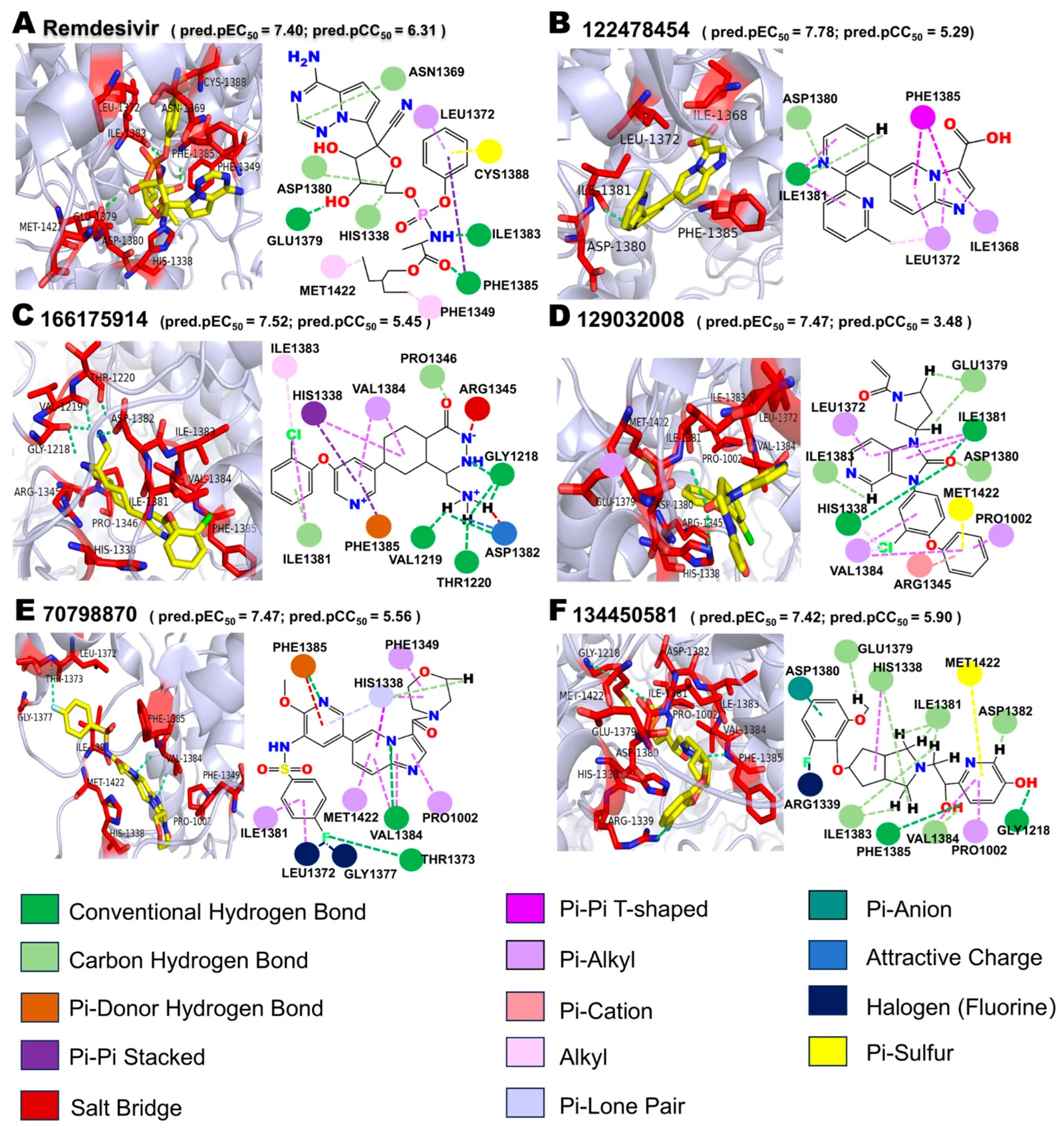
Binding modes of potential inhibitors at the allosteric pocket of RSV L-RdRp (8FPI): (**A**) Remdesivir, (**B**) **122478454**, (**C**) **166175914**, (**D**) **129032008**, (**E**) **70798870** and (**F**) **134450581**.

To minimize stochastic bias, molecular docking between each candidate compound and RSV RdRp (PDB ID: 8FPI) was performed in triplicate (Supplementary Material File S3). To gain mechanistic insights into ligand–protein recognition, the docking poses and intermolecular interactions of Remdesivir and the 5 potential candidate compounds were visualized and analyzed in detail. As summarized in Table 3 and Figure 10, all five candidates form stable complexes within the RdRp active pocket, with CDOCKER_INTERACTION_ENERGY below −45 kcal/mol, indicating strong binding affinity toward the target enzyme.

The reference compound Remdesivir (Figure 10A; −57.55 kcal/mol, *p*EC_50_ = 7.40, *p*CC_50_ = 6.31) establishes a diverse interaction network with RdRp. Its C-H fragments form carbon hydrogen bonds with HIS1338, ASN1369, and ASP1380. The aromatic moiety engages in π-alkyl interaction with LEU1372, π-π T-shaped stacking with PHE1385, and π-sulfur interaction with CYS1388. In addition, alkyl interactions are observed with PHE1349 and MET1422, while the amino, carbonyl, and hydroxyl groups form conventional hydrogen bonds with ILE1383, PHE1385, and GLU1379, respectively. These interactions collectively stabilize Remdesivir within the catalytic pocket.

Compound **122478454** (Figure 10B; −45.00 kcal/mol, *p*EC_50_ = 7.78, *p*CC_50_ = 5.29) forms a highly organized interaction network dominated by heteroaromatic recognition. Its nitrogen-containing heterocycle forms π-alkyl interactions with ILE1368 and LEU1372. The imidazo[1,2-a]pyridine core also produces a π-π T-shaped stacking interaction with PHE1385. ILE1381 plays a key role by forming multiple interactions at the same time. It participates in π-alkyl binding, conventional hydrogen bonding with the pyridine nitrogen, and carbon hydrogen bonding with C-H groups. ASP1380 further strengthens the binding through carbon hydrogen bonds. These combined interactions contribute to the high biological activity of the compound.

Compound **166175914** (Figure 10C; −72.61 kcal/mol, *p*EC_50_ = 7.52, *p*CC_50_ = 5.45) exhibits the strongest docking affinity among all candidates. The amino group forms several classic hydrogen bonds with GLY1218, VAL1219, THR1220, and ASP1382, and also shows an attractive charge interaction with ASP1382. The ring nitrogen forms a salt bridge with ARG1345. Moreover, π-alkyl and π-π stacking interactions with HIS1338 and VAL1384, alkyl contacts with ILE1383, π-donor hydrogen bonds with ILE1381 and PHE1385, and a carbon hydrogen bond with PRO1346 together build a tight and highly cooperative binding network.

Compound **129032008** (Figure 10D; −54.95 kcal/mol, *p*EC_50_ = 7.47, *p*CC_50_ = 3.48) interacts with L-RdRp through extensive hydrophobic and polar contacts. Its cyclic structure forms π-alkyl interactions with PRO1002, LEU1372, ILE1381, and VAL1384. The chlorine substituent also forms alkyl interactions with HIS1338 and VAL1384. The carbonyl group forms both classic and carbon hydrogen bonds with ILE1381, HIS1338, ASP1380, and GLU1379. Binding stability is further enhanced by a π-cation interaction with ARG1345 and a π-sulfur interaction with MET1422.

Compound **70798870** (Figure 10E; −63.40 kcal/mol, *p*EC_50_ = 7.47, *p*CC_50_ = 5.56) generates a wide hydrophobic network with PRO1002, HIS1338, PHE1349, LEU1372, ILE1381, VAL1384, and MET1422. HIS1338 acts as a key residue by forming π-π stacking, π-lone pair, and carbon hydrogen bonds. The fluorine atom forms halogen bonds with LEU1372 and GLY1377, plus a hydrogen bond with THR1373. Extra hydrogen bonds and π-donor interactions with PHE1385 further stabilize the binding conformation.

Compound **134450581** (Figure 10F; −67.20 kcal/mol, *p*EC_50_ = 7.42, *p*CC_50_ = 5.90) displays a well-balanced interaction profile. Its cyclic structure engages in π-alkyl interactions with PRO1002, HIS1338, and VAL1384. Several C-H groups serve as hydrogen bond donors to HIS1338, GLU1379, ILE1381, ASP1382, ILE1383, and VAL1384. The hydroxyl group forms classic hydrogen bonds with PHE1385 and GLY1218, and the fluorine atom forms a fluorine-mediated halogen bond with ARG1339. Further π-anion interactions with ASP1380 and π-sulfur interactions with MET1422 also improve binding selectivity.

**Figure 11:**
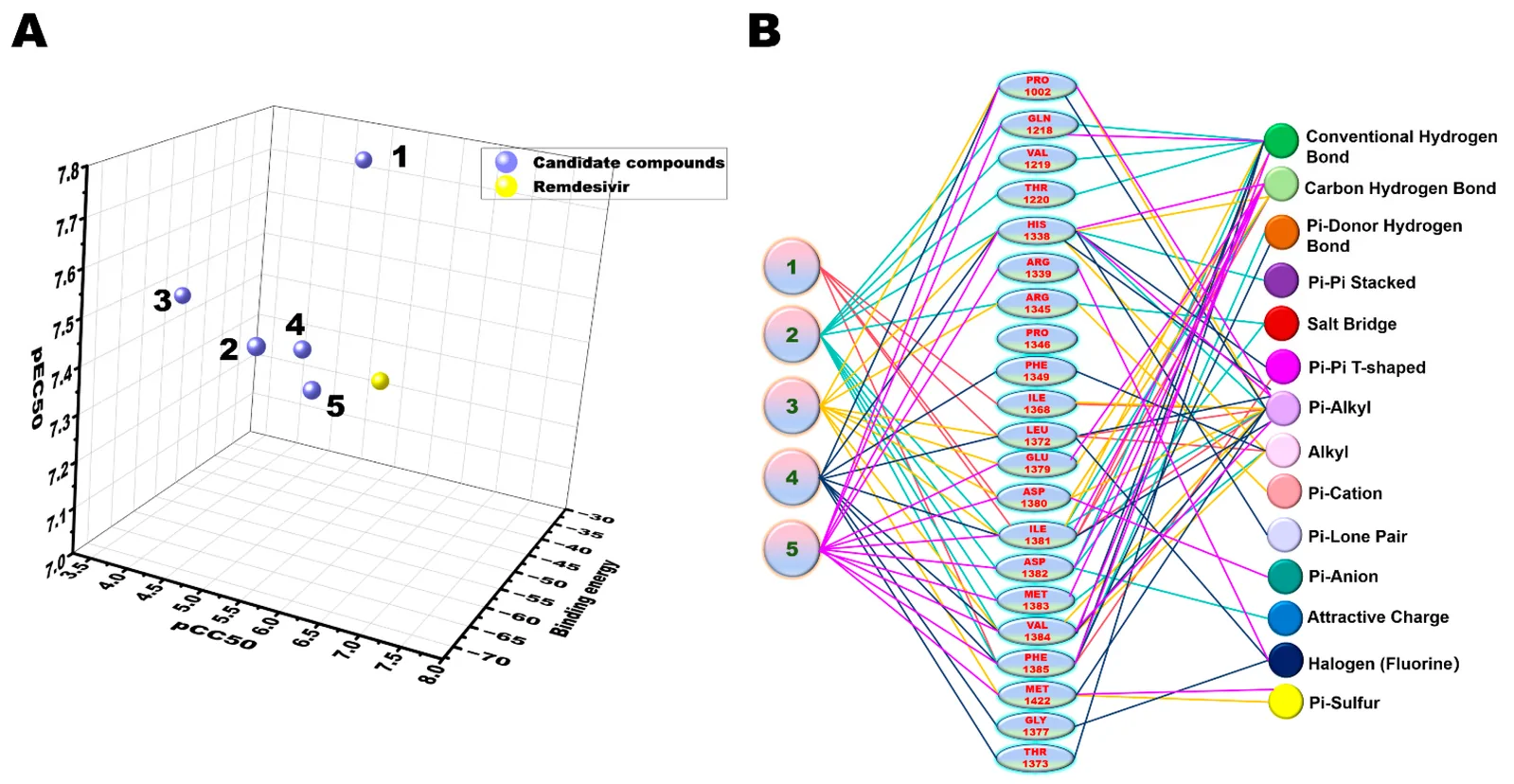
Overall performance of the five candidate RSV L-RdRp inhibitors and their binding interactions with key amino acid residues. (**A**) Binding energy, *p*CC_50_, and *p*EC_50_ values for the five candidates and reference compounds; (**B**) binding interactions between the five candidate compounds and amino acid residues of L-RdRp. The five candidate inhibitors are (1) **122478454**, (2) **166175914**, (3) **129032008**, (4) **70798870**, (5) **134450581**.

A comprehensive comparison shows that all five candidate compounds are more potent and less toxic than remdesivir. They also bind stably inside the active pocket of L-RdRp (Figure 11A). Although these compounds differ in structure, they share a common binding pattern centered on a key set of residues: HIS1338, LEU1372, ILE1381, PHE1385, and VAL1384 (Figure 11B). HIS1338 acts as a key anchoring residue involved in π-alkyl interactions, π-π stacking, and carbon hydrogen bonding. LEU1372 and ILE1381 form a stable hydrophobic framework, while PHE1385 acts as a major site for π-mediated interactions. VAL1384 enhances the binding affinity of compounds to the target through hydrophobic interactions and hydrogen bonding; residues such as ARG1345, MET1422, and ASP1380 further enhance binding stability and specificity through salt bridges, π-cation, π-sulfur, and π-anion interactions, respectively. These compounds form stable bonds with the target protein through a combination of hydrophobic interactions, polar binding, and various weak specific interactions, and exhibit strong inhibitory activity (*p*EC_50_ ≥ 7.42).

### 2.8. Molecular Dynamics Simulations of the Complexes Formed by the Top Three Candidate Inhibitors with L-RdRp (8FPI)

**Figure 12:**
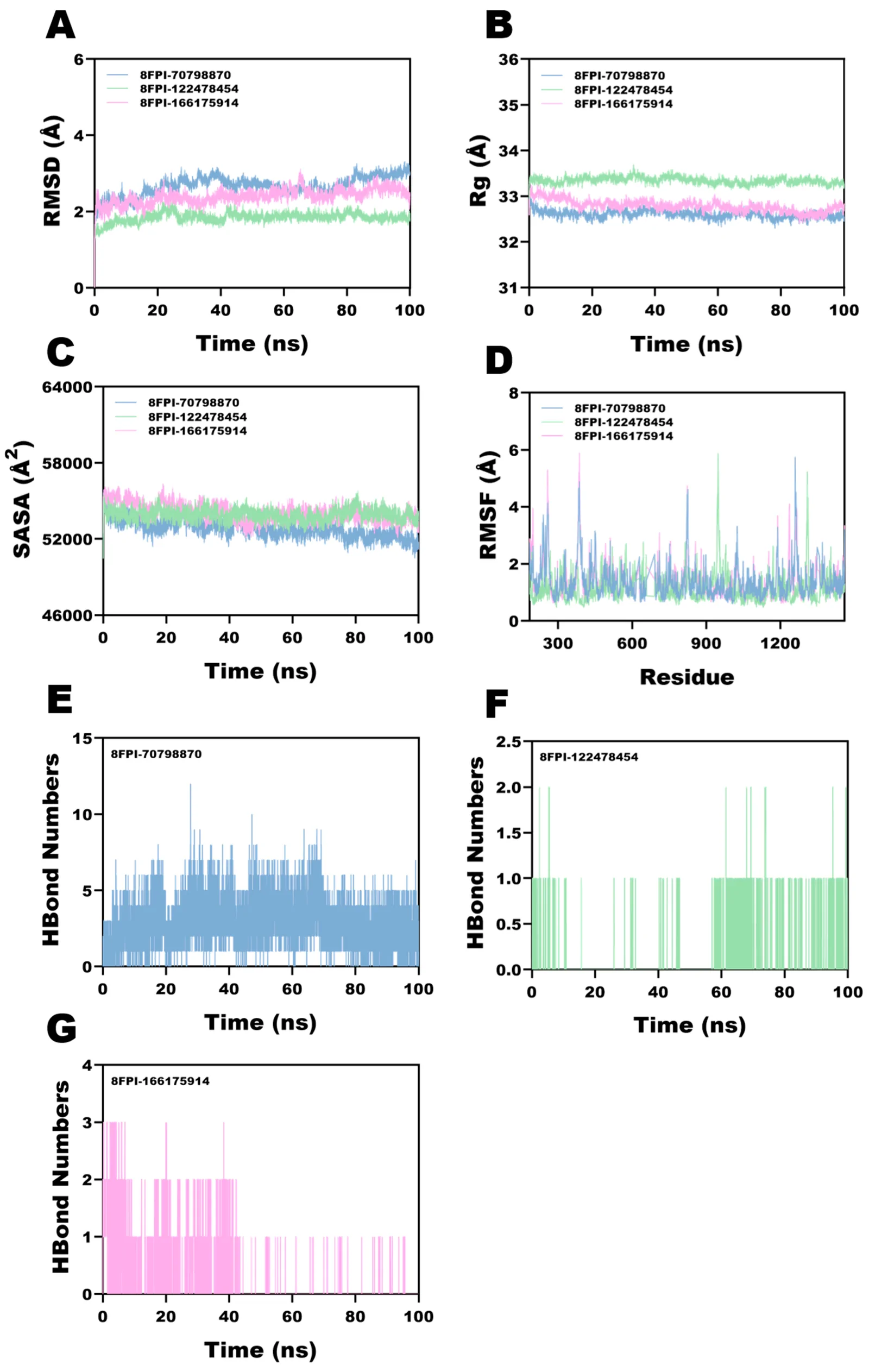
Molecular dynamics simulation of protein–ligand complexes. (**A**) RMSD values of protein–ligand complexes over time; (**B**) Rg values of protein–ligand complexes over time; (**C**) SASA values of protein–ligand complexes over time; (**D**) RMSF values of protein–ligand complexes; (**E**–**G**) HBonds values of protein–ligand complexes over time.

Root Mean Square Deviation (RMSD) is a key indicator for assessing the stability of a protein’s conformation following binding to a small-molecule ligand [44,45,46]. It is primarily used to measure the degree of deviation in atomic positions from the initial structure; the smaller the deviation, the more stable the overall conformation. Therefore, we used RMSD analysis to determine whether the molecular simulation system had reached a steady state (Figure 12A). Among these, the 8FPI-122478454 complex reached stability the fastest, stabilizing after 40 nanoseconds, with the RMSD value fluctuating around 1.8 Å. The 8FPI-166175914 and 8FPI-70798870 complexes reached equilibrium after approximately 70 ns and 80 ns, respectively, with RMSD values stabilizing at around 2.5 Å and 3.0 Å. The simulation results show that these three complexes remained stable throughout the simulation, indicating that the small molecules bind firmly to the protein and that the binding state is stable.

The radius of gyration (Rg) reflects the compactness of the protein structure [47]. As shown in Figure 12B, the Rg values for the 8FPI-122478454, 8FPI-166175914, and 8FPI-70798870 remained stable throughout the simulation, with no significant increases or decreases, indicating that the binding of the small molecules did not disrupt the overall structural integrity of the L-RdRp protein.

Solvent-accessible surface area (SASA) is commonly used to analyze changes in protein surface structure following small molecule binding [47,48]. As shown in Figure 12C, the SASA values for all three complexes exhibit no significant fluctuations, indicating that small-molecule binding does not cause significant disruption to the overall protein structure, further validating the rationality and reliability of this binding mode.

Root-mean-square flexibility (RMSF) is used to characterize the flexibility of each amino acid residue [49], a lower RMSF value indicates that the corresponding residue is more stable and exhibits smaller structural fluctuations. As displayed in Figure 12D, most residues in all three complexes had RMSF values below 6 Å. Residues in the active pocket showed especially low fluctuation. This indicates that ligand binding effectively limits local conformational changes and stabilizes key functional regions of L-RdRp.

Hydrogen bonds are essential for stabilizing protein ligand complexes. Time-dependent changes in hydrogen bond count are shown in Figure 12E–G. The 8FPI–70798870 complex had the highest hydrogen bond occupancy, ranging from 0 to 12 and remaining near six for most of the simulation. The 8FPI-122478454 and 8FPI-166175914 complexes maintained fewer hydrogen bonds, mostly around one, with ranges of 0–2 and 0–3 respectively. Even with these differences, all complexes formed stable hydrogen bond interactions that supported overall binding stability.

**Figure 13:**
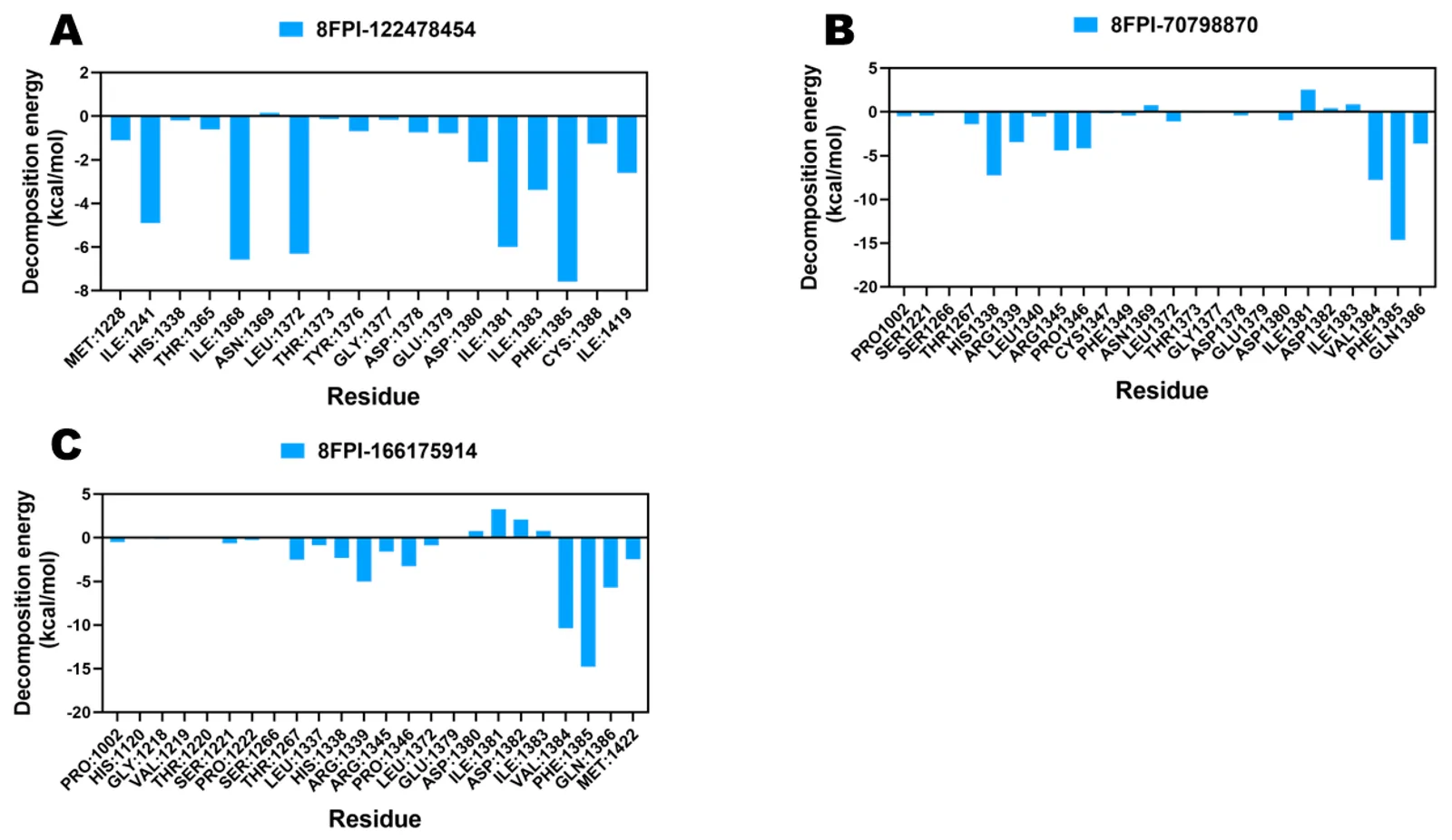
MM/PBSA combined free energy calculations. (**A**) 8FPI-122478454; (**B**) 8FPI-70798870; (**C**) 8FPI-166175914.

Using the binding conformations of the complexes, the binding free energies of the small molecules to the target protein were calculated using the MM/PBSA method. The binding free energies for the 8FPI-122478454, 8FPI-70798870, and 8FPI-166175914 complex systems were −32.890 kcal/mol, −61.046 kcal/mol, and −47.437 kcal/mol, respectively. Subsequently, this study further calculated and analyzed the amino acids that make significant contributions to small-molecule binding in the complex systems. The results showed that in the 8FPI-122478454 complex system, residues such as ILE1241, ILE1368, LEU1372, ILE1381, and PHE1385 exhibited high contribution values (Figure 13A). In the 8FPI-70798870 complex, residues such as HIS1338, VAL1384, PHE1385, and GLN1386 exhibited high contribution values (Figure 13B). In the 8FPI-166175914 complex, residues such as HIS1338, ARG1339, VAL1384, PHE1385, and GLN1386 exhibit high contribution values (Figure 13C). This suggests that these amino acid residues may play an important role in the catalytic process. Consistent binding hotspots were observed in the results of molecular docking and MM/PBSA residue energy decomposition. Key residues such as PHE1385, HIS1338, VAL1384, and ILE1381 were predicted to be involved in ligand recognition in rigid docking and were further validated under dynamic simulation conditions in aqueous solution, indicating that these amino acids serve as important small-molecule anchoring sites within the active pocket of L-RdRp. At the same time, discrepancies were observed between the affinity rankings derived from CDOCKER interaction energies and those from MM/PBSA binding free energies. This discrepancy stems from the fact that rigid molecular docking does not account for solvation effects or protein conformational flexibility. Compound **166175914** achieved the highest score in static docking, but its binding potential was reduced by the unfavorable de-solvation cost in aqueous solution; in contrast, compound **70798870** maintains excellent binding ability in both static docking and dynamic MM/PBSA evaluations, making it the most promising lead molecule among the three. Molecular docking can provide preliminary information on binding modes, while molecular dynamics combined with free energy calculations is an indispensable follow-up validation method for screening reliable candidate compounds. In summary, the 8FPI-70798870, 8FPI-122478454, and 8FPI-166175914 complex systems exhibit stable binding, and the complexes display strong hydrogen bonding. Therefore, the small molecules 70798870, 122478454, and 166175914 bind well to the 8FPI target protein.

## 3. Discussion

In addition, these five compounds have previously been reported to exert inhibitory activity against targets implicated in other diseases, illustrating the strategy for repositioning of previously reported bioactive compounds [50,51,52,53,54]. They were originally designed to modulate human disease-related targets, and their pharmacological potential has been evaluated in preclinical investigations. Compound **134450581** acts as an NR2B receptor modulator investigated for depression and neurodegenerative disorders [50]; Compound **122478454** functions as a GDF-8/TGF-β pathway modulator targeting conditions associated with muscle atrophy and fibrosis [51]; Compound 166175914 is a PRMT5 inhibitor developed for MTAP-deleted solid tumors [52]; Compound **129032008** represents a reversible BTK inhibitor studied for B-cell malignancies and autoimmune inflammation [53]; and Compound **70798870** selectively inhibits PI3Kα to suppress proliferation of mutation-driven tumors [54]. Previous studies have confirmed that these five classes of heterocyclic core structures feature simple synthetic routes, flexible side-chain modifications, and manageable preliminary toxicity, providing a solid foundation for drug development. However, no existing published data have investigated their effects on the RSV L-RdRp. This study, combining QSAR predictions, molecular docking, and MDS, demonstrates for the first time that these five molecules can stably bind to the catalytic domain of the RSV polymerase. Based on the physicochemical, synthetic, and drug development advantages reported in previous studies, these five candidate molecules have fundamental value for in vitro antiviral testing (the detailed multi-stage virtual screening statistics are summarized in Table S7 in Supplementary Material File S1).

Nevertheless, it should be acknowledged that this study has some inherent limitations. The QSAR model was trained using a small dataset derived from a single literature source and covers only L-RdRp inhibitors with a single scaffold, which limits the applicability domain of reliable predictions. Furthermore, all conclusions drawn in this paper regarding activity, safety, and binding affinity are purely computational in nature; systematic in vitro and in vivo biological experiments are still required to comprehensively validate the actual antiviral efficacy and safety of the screened candidate molecules. In future studies, we plan to expand the dataset of RSV L-RdRp inhibitors to include multiple skeletal structures, thereby broadening the covered chemical space and enhancing the generalizability of the QSAR model, followed by comprehensive biological validation of promising lead compounds.

## 4. Materials and Methods

### 4.1. Data Collection and Descriptor Calculation

A total of 75 small molecules with anti-RSV activity were retrieved from the published literature [30]. These compounds are L-RdRp protein inhibitors that target the catalytic subunit of the RSV RdRp complex. In parallel, 51 RSV L-RdRp inhibitors with experimentally reported cytotoxicity data were selected to establish a separate 2D-QSAR model for cytotoxicity prediction (Supplementary Material File S4) [30]. Notably, all inhibitors were collected from one single literature source, which inevitably narrows the covered chemical space and lowers the overall statistical power of the two QSAR models.

In accordance with the “well-defined endpoint” requirement outlined in Principle 1 of the OECD QSAR guidelines [39], we measured antiviral activity using the half-maximal effective concentration (EC_50_), that is the compound concentration that stops 50% of viral replication. For cytotoxicity, we relied on the half-maximal cytotoxic concentration (CC_50_): the concentration that kills 50% of host cells or leads to noticeable toxic effects. To improve the clarity of the data and satisfy the normality distribution assumption required for modeling [39], the original EC_50_ and CC_50_ (μM) were converted to molar concentration (M) and subsequently transformed using a logarithmic scale to obtain *p*EC_50_ (−logEC_50_) and *p*CC_50_ (−logCC_50_). In this study, higher *p*EC_50_ values represent stronger antiviral activity, while higher *p*CC_50_ values indicate greater cytotoxicity [55,56].

All compounds were structurally drawn and verified using ChemDraw 22.2.0. Molecular descriptors were generated using AlvaDesc 2.0.16 [57] and PaDEL 2.21 [58]. There are natural differences between 2D and 3D descriptors. 3D descriptors usually require the determination of stable conformers or conformational sets, which can raise computational complexity and lower the reproducibility of models [59]. For this reason, this study adopted 2D descriptors with clear physicochemical significance and stable calculation performance to improve model transferability, reproducibility and interpretability [60]. Descriptors with a correlation coefficient |R| greater than 0.95 and those with missing values were excluded. We imported the leftover descriptors into QSARINS 2.2.4 to build the model [36]. For untested external compounds, we extracted and preprocessed their descriptors the same way.

### 4.2. Dataset Splitting and Model Development

To make the model more stable and check how reliable its predictions are, we used the following three ways to split the dataset: grouping by activity values, grouping by molecular structure, and random splitting [36]. The training set and test set were divided at ratios of 3:1 and 4:1. Full details can be found in Supplementary Material File S4.

After calculating the molecular descriptors, we used a genetic algorithm to pick out the most important features. This method follows OECD QSAR Principle 2, which demands that the algorithm used be clear and easy to understand [39]. The final QSAR model was built using MLR in the QSARINS 2.2.4 software [36]. During the modeling process, the QUIK value was set to 0.05 to prevent multicollinearity issues among molecular descriptors [29,61]. To balance computational efficiency with predictive accuracy and avoid overfitting, the genetic algorithm (GA) was configured with a mutation rate of 50%, 200 generations, and a population size of 2000. Furthermore, to minimize spurious correlations, we followed a common rule of thumb to ensure that the ratio of the number of compounds in the training set to the number of selected descriptors was no less than 5 [36]. Subsequently, the optimal model was identified by evaluating several validation metrics and applied to subsequent virtual screening.

### 4.3. Model Validation, Screening, and Evaluation

To ensure our QSAR model is reliable and usable, we fully evaluated its fitness, stability, and predictive ability following Principle 4 of the OECD QSAR Guidelines [39]. In this study, we used both internal and external validation to thoroughly check the model’s robustness, reliability, and practical performance [62].

Internal validation is primarily used to assess the model’s stability on the training set. Common metrics include the coefficient of determination *R*^2^, the adjusted coefficient of determination $R_{adj}^{2}$, and the leave-one-out cross-validation coefficient $Q_{LOO}^{2}$. Additionally, Y-randomization tests are employed to rule out spurious correlations; qualified models exhibit a significant decrease in $Q_{Yscr}^{2}$ and $R_{Yscr}^{2}$ following randomization [63,64].

External validation, on the other hand, is used to assess the model’s predictive capability and generalization ability for new compounds. It primarily employs the external cross-validation coefficient $Q_{Fn}^{2}$ and the consistency correlation coefficient *CCC*_test_ to evaluate the stability and consistency of predictions [37], and uses the root mean square error *RMSE*_test_ and mean absolute error *MAE*_test_ to measure predictive accuracy [38]. Furthermore, $r_{m(test)}^{2}$ and ${\Delta r}_{m(test)}^{2}$ are used to determine whether the distribution of prediction errors is uniform, thereby avoiding systematic errors [65].

This study employs a multi-criteria decision-making method to optimize QSAR models [36]. The selected models must meet a series of strict criteria as follows: *R*^2^ > 0.70, $Q_{LOO}^{2}$ > 0.60, $Q_{Fn}^{2}$ > 0.70, *CCC*_test_ > 0.85, $r_{m(test)}^{2}$ > 0.65, ${\Delta r}_{m(test)}^{2}$ < 0.20, and low *MAE*_test_ and *RMSE*_test_ values [37,66]. This thorough validation step makes sure the model fits data well, works reliably on new compounds, and gives trustworthy prediction results. Detailed descriptions of each validation metric are provided in Table S8 of Supplementary Material File S1.

### 4.4. Mechanism Interpretation and Evaluation of the Model’s Applicability Domain

This study followed the third principle of the OECD QSAR guidelines, which requires a clear applicability domain. A double threshold method was used to check structural and response outliers. This method controls two main sources of uncertainty. It limits deviations from the training chemical space and reduces large prediction errors. Leverage values were used to find structural outliers. The threshold was set as $h^{*}=3(p+1)/n$. In this formula, *p* represents the number of descriptors, and n represents the number of compounds in the training set. Compounds with a leverage value greater than $h^{*}$ are considered outside the model’s chemical space, so their predictions are not reliable [67]. Response outliers were judged by standardized residuals. Compounds with residuals over 3σ were excluded, where σ is the residual standard deviation [36,67]. The applicability domain was shown by Williams plot. The acceptable area is limited by h* and ±3σ. Compounds outside this area were not within the model scope. We also used an Insubria plot to visually demonstrate the model’s ability to predict the activity of external real-world compounds. This plot allows us to verify whether the structural features of these compounds fall within the reasonable range of the training set, thereby enhancing the reliability of the virtual screening results.

In accordance with Principle 5 of the OECD QSAR Guidelines, predictive models must explain the underlying biological mechanisms as clearly as possible [39]. Therefore, we interpreted the model’s mechanism of action through the analysis of key molecular descriptors, molecular feature mapping, and structure–activity and structure–cytotoxicity relationships. We used VIP analysis to identify important molecular descriptors, defining those with VIP values greater than 1 as primary influencing factors [47]. We then utilized descriptor load plots to visually demonstrate how these features individually influence compound activity and cytotoxicity, mapping them to specific chemical structures to explain their relationship with biological effects. Concurrently, we compared representative compounds with high/low activity and high/low cytotoxicity to clarify the impact of different structural fragments on antiviral efficacy and safety. This comprehensive mechanistic analysis provides a scientific basis for the design and optimization of RSV L-RdRp inhibitors with enhanced efficacy and improved safety.

### 4.5. Virtual Screening Using Optimal Models and Molecular Docking

The primary purpose of constructing a 2D-QSAR model is to conduct virtual screening of potential RSV L-RdRp inhibitors. Such models can directly predict the antiviral activity and cytotoxicity of external compounds without experimental data, helping us rapidly identify high-activity, low-cytotoxicity candidate molecules from massive compound libraries. This not only significantly improves screening efficiency but also prioritizes the selection of compounds with drug-like potential for subsequent experimental validation. Restricted by the aforementioned limitations of small sample size, single data source and narrow chemical space coverage of the training sets, the predictive range of the models is limited, and all predicted results are only reliable within the AD of the training set.

In this study, remdesivir was used as the screening reference [33]. We first collected over 50,000 external compounds with a bis-N-heterocyclic conjugated core scaffold from the PubChem and ChEMBL databases (Supplementary Material File S1, Table S9). After excluding salts, complexes, overly large molecules, and compounds already present in the modeling dataset, we ultimately obtained 15,758 molecules for subsequent virtual screening. We calculated molecular descriptors for these compounds and the reference drug remdesivir and input the key descriptors into optimized activity and cytotoxicity QSAR models to obtain predictive results. We also employed a predictive reliability index tool to classify prediction reliability into three levels, “Good”, “Medium”, and “Bad”, in order to assess the model’s reliability in predicting the actual activity of external compounds [68,69]. To ensure the reliability of the results, we retained only those compounds that fell within the model’s applicability range and exhibited superior inhibitory effects compared to remdesivir.

Molecular docking, as a structure-based screening step, is used to screen compounds prioritized by the QSAR model. The cryo-EM structure of the RSV RdRp complex bound to the allosteric inhibitor MRK-1 (PDB ID: 8FPI) was imported into Discovery Studio. In this structure, the resolved ligand-binding pocket is located within the domain of the L protein [30,70]. Consequently, retaining only the L protein as the docking receptor and redundant heteroatoms and ions were removed. Polar hydrogen atoms were added to the RSV L protein chain, the CHARMM force field was assigned, and energy minimization was carried out to optimize protein geometry for subsequent docking calculations. Retaining only the L protein chain preserves the core inhibitor-binding pocket while reducing the computational cost of large-scale virtual screening. All small-molecule compounds were processed via the Prepare Ligand module in Discovery Studio. Protonation states were assigned at physiological pH 7.4, and initial geometry optimization was performed to obtain stable low-energy conformations before docking. However, this simplified receptor model has some limitations. Retaining only the L-protein chain as the docking template eliminates inter-subunit interactions and conformational constraints that may influence the local geometry and dynamics of the L-protein. Therefore, the docking analysis in this study serves only as a structure-based filtering step for compounds identified through QSAR screening against L-RdRp, rather than as a complete representation of ligand binding within the full L-p holoenzyme. We then processed the structure by removing water molecules and ions and adding polar hydrogen atoms. The binding pocket was defined according to the coordinates of the original ligand (X = 113.83 Å, Y = 97.58 Å, Z = 137.12 Å; radius = 11.2 Å). Notably, the reference compound remdesivir is a nucleoside analog that natively targets the canonical nucleotide catalytic site. In the present work, the allosteric pocket bound by non-nucleoside inhibitor MRK-1 was uniformly adopted as the docking cavity for all screened external compounds. Simulations were not performed at remdesivir’s native catalytic pocket, and this design enables parallel evaluation of the potential binding affinity of remdesivir and external candidates at this allosteric site. By re-docking the native ligand, an RMSD value of 0.7540 Å was obtained, validating the reliability of the entire set of parameters for this docking system (RMSD value, full docking score and binding conformation of MRK-1 are provided in Supplementary Material File S5). The ligand was prepared using the “Prepare Ligand” module, and the docking calculation was performed using the CDOCKER module for preliminary screening of candidate compounds, which is a CHARMM-based rigid receptor docking algorithm [71]. During the CDOCKER calculations, the receptor protein was kept rigid, while the ligand was treated as fully flexible during conformational optimization. The “Random Conformations” parameter was set to 10, thereby generating ten independent random initial conformations for each ligand, which were used for simulated annealing and energy minimization.

Conformations were ranked and screened based on the CDOCKER_INTERACTION_ENERGY metric. This metric specifically quantifies static intermolecular non-bonded interactions between the ligand and the protein, excluding the ligand’s own intramolecular strain energy; we selected this metric because the focus of the study was on direct intermolecular contacts formed within the allosteric pocket of RSV L-RdRp, rather than conformational strain within the ligand itself. This scoring function is not equivalent to the absolute thermodynamic binding free energy; higher values merely indicate, in a qualitative sense, the presence of more favorable static binding contacts in different conformations of the same ligand, rather than providing quantitative evidence of actual binding affinities between different compounds.

To minimize random effects, each docking run was repeated three times. The optimal binding conformation for each compound was selected by comprehensively considering the highest CDOCKER_INTERACTION_ENERGY values. The final binding conformations were visualized using PyMOL 3.9 [72]. Rigid receptor docking fails to capture the large-scale flexible rearrangements of the RSV L-RdRp domain; to address this limitation, the docking conformations of the screened potential inhibitors with the protein complex were subjected to further 100 ns molecular dynamics simulations to characterize the dynamic ligand–protein binding behavior.

Given that unacceptable toxicity is one of the primary causes of drug candidate failure during development [73,74,75,76,77], cytotoxicity prediction was introduced as a critical early-stage screening criterion. Compounds predicted to exhibit high cytotoxicity were excluded prior to docking and downstream ADMET evaluation, even if their predicted inhibitory activity was favorable. This method effectively reduces the chemical range to low-cytotoxicity candidates with better development value. It also optimizes the use of computing and experimental resources and improves the efficiency and success rate of finding RSV L-RdRp inhibitors.

### 4.6. Drug-Likeness Evaluation

Drug-likeness reflects whether a compound has suitable ADMET properties. These properties include absorption, distribution, metabolism, excretion and toxicity. They are very important for later drug development [78,79]. A two-step evaluation was used to select compounds with good pharmacokinetic potential. First, the SWISS-ADME platform was used to conduct a preliminary pharmacokinetic screening of external compounds [80]. Common screening rules, including those proposed by Lipinski, Ghose, Oprea, Veber, and Muegge [34,35,81,82,83,84], were applied to identify compounds that met at least three of these criteria. In addition, gastrointestinal absorption and synthetic feasibility were evaluated to further narrow down the list of candidate compounds. In the second step, the pkCSM platform was used to conduct an in-depth ADMET analysis [85]. This step helped identify the compounds with the best overall druggability.

In this study, “boiled egg” plots and radar charts were used to clearly illustrate the drug-like properties of the compounds [80]. The vertical axis, WLOGP (Wildman–Crippen Lipophilicity), reflects molecular lipophilicity; the horizontal axis, TPSA (Topological Polar Surface Area), reflects polarity and hydrogen-bonding capacity. The white region indicates a high probability of passive gastrointestinal absorption (HIA+), suggesting high potential for oral bioavailability. The yellow region indicates a high probability of blood–brain barrier (BBB) penetration (BBB+), implying potential for central nervous system (CNS) action or a high risk of CNS side effects. The last region indicates low probabilities for both absorption and BBB penetration, indicating poor potential for oral drug development. Radar charts were used to evaluate six key drug-like properties, including lipophilicity, molecular size, polarity, solubility, flexibility, and saturation. These charts provide a clear visual indication of the drug-like potential of these compounds; this allows quick screening early in drug development.

### 4.7. Molecular Dynamics Simulations (MDS)

We employed molecular dynamics simulations (MDS) as a supplementary validation method to further verify the reliability of the molecular docking results and to investigate the dynamic stability of the protein–small molecule complex. MDS can show the flexibility, binding stability and interactions of biological molecules at the atomic level under simulated physiological conditions [86,87]. All simulations were run in GROMACS 2022. To verify whether the selected compounds could form stable complexes with RSV L-RdRp, we selected three representative compounds with high, medium, and low docking scores for further analysis. First, we generated protein topology files using the pdb2gmx tool in conjunction with the AMBER14SB force field. We then used the sobtop_1.0 software to construct ligand parameters based on the GAFF2 force field. Additionally, we employed the RESP method to calculate atomic charges, ensuring a reasonable and reliable charge distribution. Subsequently, each protein–ligand complex was placed in a cubic water box using the TIP3P water model, with the distance between the solute and the water box boundary set to 1.0 nm [88]. Na^+^ and Cl^−^ ions were added to bring the ionic concentration of the system to 0.15 M, simulating the human physiological environment while maintaining the overall electrical neutrality of the system. For the computational method, we used the Particle-Mesh Ewald (PME) method to handle long-range electrostatic interactions, with a cutoff distance set to 1.0 nm, and constrained bond lengths using the LINCS algorithm. Prior to the main simulation, the system underwent energy minimization. Structural optimization was performed using 3000 steps of the steepest descent method followed by 2000 steps of the conjugate gradient method to eliminate unreasonable spatial conflicts between atoms. Subsequently, a 100 ns molecular dynamics simulation was performed under the NPT ensemble, with a time step of 2 fs. During the simulation, the temperature was stabilized at 310 K using a Nosé–Hoover thermal bath, while the pressure was controlled at 1 bar via a Parrinello–Rahman pressure bath.

During the simulation process, the gmx rmsd, gmx rmsf, gmx hbond, gmx gyrate, gmx sasa, and gmx sham tools were used to calculate the root-mean-square deviation (RMSD), root-mean-square fluctuation (RMSF), number of hydrogen bonds, gyration radius (Rg), and solvent-accessible surface area (SASA), respectively. The MM-PBSA binding free energy of the complex was calculated using gmx_mmpbsa. This was done to analyze the system’s stability, structural changes, and solvent effects [89,90,91,92].

## 5. Conclusions

To address the current lack of effective 2D-QSAR-based virtual screening strategies for RSV L-RdRp inhibitors, this study developed a multidimensional computer-aided drug discovery framework that integrates activity, cytotoxicity, druggability, and good pocket compatibility. Using this comprehensive strategy, five potential hit compounds targeting RSV L-RdRp (CID: **122478454**, **166175914**, **129032008**, **70798870**, and **134450581**) were identified, providing valuable theoretical guidance for the development of anti-RSV drugs. First, robust 2D-QSAR models for predicted inhibitory activity (*p*EC_50_) and cytotoxicity (*p*CC_50_) were constructed in strict accordance with OECD principles. Comprehensive internal and external validation confirmed their strong goodness-of-fit, robustness, and predictive reliability. Mechanistic analysis showed that compounds with abundant pyridine frameworks and beneficial electronic and topological features such as nPyridines and VE2sign_B(s) tend to exhibit higher inhibitory potency within the QSAR model. Unfavorable topological connections between atom pairs such as F05[N-Cl] and F07[C-N] show the opposite effect and weaken compound activity. Cytotoxicity is mainly associated with the distribution of nitrogen atoms, molecular rigidity, and long-range topological descriptors. These results can help us translate complex structure–activity relationships into simple, practical methods for molecular design and optimization. At the same time, we utilize the multidimensional computer-aided drug discovery screening framework we have established, ultimately identifying five high-quality candidate molecules from a library of 15,758 external compounds. Compared to remdesivir, these candidate compounds demonstrate higher predicted inhibitory activity, lower predicted cytotoxicity, better oral bioavailability, and a more stable predicted binding mode to the target. Molecular docking and dynamics simulation results indicate that these compounds stably bind to the conserved residues HIS1338, LEU1372, ILE1381, PHE1385, and VAL1384 on the L-RdRp via hydrophobic, polar, and various specific interactions. Furthermore, key metrics such as RMSD, Rg, and SASA remained stable throughout the 100 ns simulation. Additionally, these five compounds have previously been reported to exert inhibitory activity against targets related to other diseases, further supporting the strategy of exploring their anti-RSV potential via repositioning of previously reported bioactive compounds.

Overall, this study has established a reliable and reusable QSAR-based virtual screening paradigm and has provided promising lead candidates for subsequent experimental validation, structural optimization, and the translational development of next-generation anti-RSV therapeutics.

## Figures and Tables

**Table 1:** Validation statistics for predicting inhibitory activity and cytotoxicity of RSV L-RdRp inhibitors.

Internal Validation Metrics					External Validation Metrics								
$R^{2}$	${RMSE}_{tr}$	${MAE}_{tr}$	$Q_{LOO}^{2}$	R^2^-$Q_{LOO}^{2}$	${RMSE}_{test}$	${MAE}_{test}$	$R_{test}^{2}$	$Q_{F1}^{2}$	$Q_{F2}^{2}$	$Q_{F3}^{2}$	${CCC}_{test}$	$r_{m}^{2}$	${\Delta r}_{m}^{2}$
*p*EC_50_ Model (Rnd-MLR): ***p*EC_50_** = 2.707 + 1259.5307VE2sign_B(s) + 5.2727MATS5s + 0.0185P_VSA_charge_3 + 0.6408nPyridines − 0.3938F05[N-Cl] − 0.1464F07[C-N] + 0.0252BCUTw-1h													
0.8281	0.2891	0.2461	0.7653	0.0628	0.2378	0.1966	0.8713	0.8609	0.8594	0.8837	0.9301	0.8144	0.0341
*p*CC_50_ Model (Rnd-MLR): ***p*CC_50_** = 6.2224 + 0.1367VE3sign_Dz(v) + 0.6596GATS5e + 0.0188T(N..N) − 1.2756MDEN-22 − 1.5969MDEN-33 − 1.0513AATSC7s − 0.0875C2SP2													
0.8263	0.2031	0.1557	0.7422	0.0841	0.2120	0.1685	0.8951	0.8530	0.8380	0.8108	0.9081	0.7210	0.1292

**Table 2:** ADMET properties of candidate RSV L-RdRp inhibitors.

**ADMET**	**Property/** **Parameter**	**Value Range**		**Candidates**	
			**Remdesivir [33]**	**122478454**	**166175914**
**Absorption**	Water solubility (log mol/L)	>> *	−2.942	−2.892	−3.476
	Caco2 permeability	>> *	0.485	1.333	0.654
	Intestinal absorption (human)	≥0.8	0.713	0.973	0.975
	Skin permeability (log Kp)	<−2.5	−2.735	−2.735	−2.735
	P-glycoprotein substrate	Yes/No	Yes	Yes	Yes
	P-glycoprotein I/II inhibitor	Yes/No	No/Yes	No/No	Yes/Yes
**Distribution**	VDss (human)	>0.45	1.015	−0.011	0.321
	Fraction unbound (human)	0.01~0.90	0.115	0.402	0.177
	BBB permeability	<−1	−2.094	−1.043	−1.346
	CNS permeability	<−3	−3.937	−2.861	−0.344
**Metabolism**	CYP2D6/CYP3A4 substrate	Yes/No	No/Yes	No/No	No/Yes
	CYP1A2/CYP2C19/CYP2C9/CYP2D6/CYP3A4 inhibitior	Yes/No	No	No	No/Yes
**Excretion**	Total clearance	>> *	0.179	0.441	0.622
	Renal OCT2 substrate	Yes/No	No	No	No
**Toxicity**	AMES toxicity	Yes/No	No	No	No
	hERG I/II inhibitor	Yes/No	No/Yes	No/No	No/Yes
	Hepatotoxicity	Yes/No	Yes	Yes	Yes
	Skin sensitization	Yes/No	No	No	No
**Drug-likeness**		Yes/No	No	Yes	Yes
**ADMET**	**Property/** **Parameter**	**Value Range**		**Candidates**	
			**129032008**	**70798870**	**134450581**
**Absorption**	Water solubility (log mol/L)	>> *	−3.026	−2.906	−3.321
	Caco2 permeability	>> *	1.390	1.657	1.228
	Intestinal absorption (human)	>30%	0.933	0.880	0.930
	Skin permeability (log Kp)	<−2.5	−2.735	−2.735	−3.009
	P-glycoprotein substrate	Yes/No	Yes	Yes	Yes
	P-glycoprotein I/II inhibitor	Yes/No	Yes/Yes	Yes/No	Yes/No
**Distribution**	VDss (human)	>0.45	0.246	0.384	0.789
	Fraction unbound (human)	0.01~0.90	0.242	0.301	0.220
	BBB permeability	<−1	−0.949	−1.131	−1.280
	CNS permeability	<−3	−2.317	−3.249	−2.997
**Metabolism**	CYP2D6/CYP3A4 substrate	Yes/No	No/Yes	No/Yes	No/Yes
	CYP1A2/CYP2C19/CYP2C9/CYP2D6/CYP3A4 inhibitior	Yes/No	No/Yes	No/Yes	No
**Excretion**	Total clearance	>> *	0.39	0.249	0.697
	Renal OCT2 substrate	Yes/No	Yes	No	No
**Toxicity**	AMES toxicity	Yes/No	No	No	No
	hERG I/II inhibitor	Yes/No	Yes/No	No/Yes	No/Yes
	Hepatotoxicity	Yes/No	No	Yes	Yes
	Skin sensitization	Yes/No	No	No	No
**Drug-likeness**		Yes/No	Yes	Yes	Yes

>> *: *means higher value is better for candidate drugs.*

**Table 3:** Molecular docking outcomes, predicted activity and cytotoxicity of potential inhibitors, along with remdesivir as the reference compound.

Comp. (CID)	SMILES	CDOCKER_INTERACTION_ENERGY (kcal/mol)	Interacting Residues	Interacting Forces	*p*EC_50_/*p*CC_50_
**Remdesivir** **(Ref. comp)**	O[C@H]1[C@@](C2=CC=C3N2N=CN=C3N)(C#N)O[C@H](CO[P@](OC4=CC=CC=C4)(N[C@@H](C)C(OCC(CC)CC)=O)=O)[C@@]1([H])O	−57.55	HIS1338 PHE1349 ASN1369 LEU1372 GLU1379 ASP1380 ILE1383 PHE1385 CYS1388 MET1422	Conventional Hydrogen Bond, Carbon Hydrogen Bond, Pi-Pi T-shaped, Pi-Sulfur, Alkyl, Pi-Alkyl	7.40/6.31
**122478454**	CC1=NC(=CC=C1)C2=C(C=CC=N2)C3=CN4C(=NC=C4C(=O)O)C=C3	−45.00	ILE1368 LEU1372 ASP1380 ILE1381 PHE1385	Conventional Hydrogen Bond, Carbon Hydrogen Bond, Pi-Pi T-shaped, Alkyl, Pi-Alkyl	7.78/5.29
**166175914**	C1CC2C(CC1C3=CC(=CN=C3)OC4=CC=CC=C4Cl)C(NNC2=O)CN	−72.61	GLY1218 VAL1219 THR1220 HIS1338 ARG1345 PRO1346 ILE1381 ASP1382 ILE1383 VAL1384 PHE1385	Conventional Hydrogen Bond, Carbon Hydrogen Bond, Attractive Charge, Salt Bridge, Alkyl, Pi-Alkyl, Pi-Pi Stacked, Pi-Donor Hydrogen Bond	7.52/5.45
**129032008**	C=CC(=O)N1CC[C@H](C1)N2C3=C(C=NC=C3)N(C2=O)C4=CC(=C(C=C4)OC5=CC=CC=C5)Cl	−54.95	PRO1002 HIS1338 ARG1345 LEU1372 GLU1379 ASP1380 ILE1381 ILE1383 VAL1384 MET1422	Conventional Hydrogen Bond, Carbon Hydrogen Bond, Pi-Sulfur, Alkyl, Pi-Alkyl, Pi-Cation	7.47/3.48
**70798870**	COC1=C(C=C(C=N1)C2=CN3C(=NC=C3C(=O)N4CCOCC4)C=C2)NS(=O)(=O)C5=CC=C(C=C5)F	−63.40	PRO1002 HIS1338 PHE1349 LEU1372 THR1373 GLY1377 ILE1381 VAL1384 PHE1385 MET1422	Conventional Hydrogen Bond, Carbon Hydrogen Bond, Pi-Donor Hydrogen Bond, Pi-Alkyl, Pi-Pi T-shaped, Pi-Lone Pair, Halogen (Fluorine)	7.47/5.56
**134450581**	COC1=C(C(=CC=C1)F)OC2C[C@@H]3CN(C[C@@H]3C2)CC(C4=NC=C(C=C4)O)O	−67.20	PRO1002 GLN1218 HIS1338 ARG1339 ASP1382 MET1383 ILE1381 VAL1384 PHE1385 ASP1380 GLU1379 MET1422	Conventional Hydrogen Bond, Carbon Hydrogen Bond, Pi-Alkyl, Pi-Sulfur, Pi-Anion, Halogen (Fluorine)	7.42/5.90

## Data Availability

Data will be made available on request.

## Supplementary Materials

The following supporting information can be downloaded at https://www.mdpi.com/article/10.3390/ph19081142/s1, Supplementary Material File S1, Tables S1–S9; Supplementary Material File S2, ADMET results; Supplementary Material File S3, the five candidates-L-RdRp docking result visualization; Supplementary Material File S4, Modeling compounds data; Supplementary Material File S5, MRK-1&8FPI docking result. Refences [93,94,95,96,97,98,99,100,101,102] are cited in Supplementary Materials.
